# Oxidative stress driven airway mesenchymal reprogramming in asthma: mechanistic insights and evidence from botanical drug formulations

**DOI:** 10.3389/fphar.2026.1770036

**Published:** 2026-03-19

**Authors:** Ling Rao, Ting Wang, Miaofen Zhang, Huiting Huang, Zhiyan Luo, Wujin Wen, Gang Liao, Shaofeng Zhan, Xiufang Huang, Yong Jiang

**Affiliations:** 1 The First Affiliated Hospital of Guangzhou University of Chinese Medicine, Guangzhou, China; 2 Shenzhen Hospital of Integrated Traditional Chinese and Western Medicine, Shenzhen, China; 3 Guangdong Clinical Research Academy of Chinese Medicine, Guangzhou, China; 4 Guangdong Engineering Technology Research Center of Commercialization of Lingnan Special Medical Institution Preparations, Guangzhou, China; 5 Lingnan Medical Research Centre of Guangzhou University of Chinese Medicine, Guangzhou, China; 6 Nanfang Hospital, Southern Medical University, Guangzhou, China; 7 State Key Laboratory of Traditional Chinese Medicine, Guangzhou, China

**Keywords:** airway mesenchymal reprogramming, asthma, botanical drugs, metabolites, oxidative stress

## Abstract

Asthma-associated airway mesenchymal reprogramming refers to a dynamic pathological process characterized by persistent pathological alterations in phenotype, function, and intercellular interactions of airway mesenchymal cells under asthmatic conditions, which actively drive airway structural changes, airway narrowing, and impaired lung function. It is considered one of the key pathological mechanisms driving the chronic progression of asthma and contributing to persistent airflow limitation. Oxidative stress, as a central pathogenic factor, drives asthma-associated airway mesenchymal reprogramming through multiple mechanisms, including promoting inflammation, inducing epithelial-to-mesenchymal transition (EMT), exacerbating airway smooth muscle dysfunction, and impairing endogenous antioxidant defense systems. Increasing evidence suggests that this reprogramming may initiate at early stages of asthma, highlighting its potential relevance as an early pathogenic indicator. Although standard therapies such as inhaled corticosteroids and β_2_-agonists are effective in controlling acute inflammation, they show limited efficacy in effectively modulating or substantially improving established mesenchymal reprogramming. Traditional Chinese Medicine (TCM), with its long history of use in asthma, offers a complementary therapeutic approach due to its multi-component, multi-target actions, low adverse effect profile, and anti-inflammatory, antioxidant, and modulatory effects on airway mesenchymal reprogramming. This review focuses on the potential of metabolites originating from botanical drugs used in TCM and botanical drug formulations in modulating oxidative stress to intervene in asthma-associated airway mesenchymal reprogramming, providing comprehensive evidence to support mechanistic investigation and translational research in managing this key pathological process.

## Graphical Abstract

**Figure:**
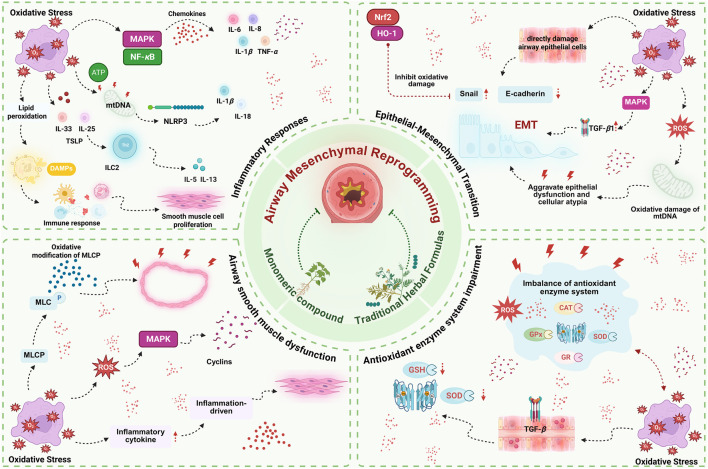

## 1 Introduction

Asthma is a chronic inflammatory airway disease in which structural remodeling contributes to persistent airflow limitation and disease progression (Mims, 2015). A key pathological basis of airway remodeling in asthma is airway mesenchymal reprogramming, a dynamic process that involves persistent phenotypic and functional alterations of structural cells within the bronchial wall. Key components include epithelial–mesenchymal transition (EMT), airway smooth muscle cells (ASMCs) and fibroblast activation, and altered crosstalk with immune cells under inflammatory and oxidative stress conditions (Zhu and Li, 2018; Sun et al., 2020). This process is accompanied by disruptions in extracellular matrix (ECM) composition and regulation, leading to sustained changes in airway structure and microenvironment that contribute to airway narrowing and functional decline (Zhou et al., 2023a; Hirota and Martin, 2013; Varricchi et al., 2024). Studies have shown that mesenchymal reprogramming can be detected even in patients with mild asthma, becomes more pronounced with increasing disease severity, and may precede detectable airway remodeling, suggesting that it reflects upstream cellular and molecular events driving later structural outcomes (Hackett et al., 2009; Hackett, 2012). Consequently, early therapeutic intervention targeting airway mesenchymal reprogramming and the development of strategies capable of preventing or effectively modulating this process are essential for improving asthma management and optimizing long-term clinical outcomes (Winkler and Frey, 2021).

Oxidative stress represents a critical pathogenic mechanism underlying asthma progression and is an important mechanistic contributor to airway mesenchymal reprogramming. It arises from an imbalance between oxidant formation (primarily reactive oxygen species, ROS, and reactive nitrogen species, RNS) and endogenous antioxidant defense capacity (Michaeloudes et al., 2022; Sies, 2020). Oxidative stress amplifies inflammatory signaling, promotes EMT, and drives airway smooth muscle (ASM) proliferation/remodeling, while also weakening endogenous antioxidant defenses, thereby contributing to airway mesenchymal reprogramming and persistent structural changes in the asthmatic airway (Michaeloudes et al., 2022).

In recent years, antioxidants such as N-acetylcysteine (NAC) have been explored to mitigate oxidative injury in asthma, yet their clinical translation remains limited and the impact on reprogramming-related outcomes is not consistently established (Mokra et al., 2023; Zhou et al., 2023b). In parallel, metabolites originating from botanical drugs and multi-component botanical drug formulations derived from traditional Chinese medicine (TCM) have been investigated for potential multi-target modulation of oxidative stress and related remodeling pathways. For example, certain flavonoids have been reported to engage Nrf2-associated cytoprotective responses and attenuate pro-remodeling signals in preclinical models (Bondonno et al., 2024), and formulations such as the Bushen Yiqi formula (BSYQF) have been linked to reduced oxidative injury and suppression of remodeling-associated mediators including transforming growth factor beta (TGF-*β*) and vascular endothelial growth factor (VEGF) (Cui et al., 2019). Although much of the existing literature focuses on airway remodeling as an outcome, these studies can be interpreted within a mesenchymal reprogramming framework that captures upstream cellular and molecular processes driving structural change (Huang and Qiu, 2022; Hough et al., 2020). Based on this perspective, this review summarizes the evidence on metabolites derived from botanical drugs and botanical drug formulations in regulating oxidative stress–driven airway mesenchymal reprogramming in asthma, and highlights the limitations of the currently available studies.

## 2 Methods

A systematic literature review was conducted by searching prominent academic databases, such as PubMed, ScienceDirect, Web of Science, Google Scholar, and the Cochrane Library. The literature search included studies published up to July 2025, aiming to capture both foundational and recent advances relevant to asthma, oxidative stress, and airway mesenchymal reprogramming. The literature strategy utilized a combination of relevant keywords, such as “asthma,” “airway mesenchymal reprogramming,” “airway remodeling,” “oxidative stress,” “traditional Chinese medicine,” and broader terms commonly used in the literature to describe plant-derived medicines (e.g., “herbal medicine” or “herbal formula”). Reference lists of relevant review articles were also manually screened to identify additional studies of potential relevance. For consistency with ethnopharmacological best practice, the term “botanical drug(s)” is used throughout this review to describe medicinal products of plant origin. Eligible studies were identified based on the following inclusion criteria: 1. peer-reviewed primary research publications; and 2. studies investigating metabolites originating from botanical drugs or botanical drug formulations relevant to asthma; and 3. studies examining oxidative stress-related mechanisms involved in airway mesenchymal reprogramming or airway remodeling. Exclusion criteria included: 1. publications without accessible full texts; 2. articles not published in English; and 3. studies not directly aligned with the core objectives of the review, including studies reporting metabolites supported only by *in vitro* assays or those with potential false-positive/assay-interference liabilities, which were excluded to reduce the risk of non-specific/false-positive signals. The included studies were reviewed with attention to study design, relevance of experimental models, and outcome measures related to oxidative stress and airway remodeling or mesenchymal reprogramming. Given the narrative scope of this review and the heterogeneity of the available studies, no formal meta-analysis was conducted.

## 3 Mechanisms of oxidative stress mediating airway mesenchymal reprogramming in asthma

### 3.1 Sources and mechanisms of oxidative and nitrosative stress in asthma-associated airway mesenchymal reprogramming

**FIGURE 1:**
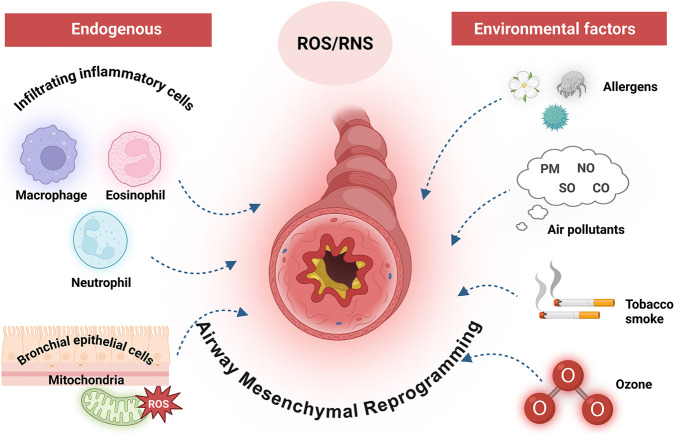
Endogenous and environmental sources of ROS/RNS in asthmatic airways. Endogenous ROS/RNS production is primarily mediated by infiltrating inflammatory cells (macrophages, eosinophils, and neutrophils), bronchial epithelial cells, and mitochondria. Environmental factors, including allergens, air pollutants, tobacco smoke, and ozone, further exacerbate oxidative stress. This enhanced oxidative environment contributes to the amplification of airway inflammation and drives airway mesenchymal reprogramming, a key process underlying early structural and functional changes in asthmatic airways. Figure created with BioRender.com.

ROS and RNS are chemically reactive molecules continuously generated during physiological metabolic activities as well as in response to environmental stimuli. Under physiological conditions, ROS and RNS are integral to maintaining cellular signaling and homeostatic balance. However, when present in excess, these reactive species inflict substantial oxidative injury on vital biomolecules such as lipids, proteins, and nucleic acids. This molecular injury is closely linked to both the initiation and exacerbation of asthma pathogenesis (Pan et al., 2025a; Santibáñez et al., 2025). In asthmatic individuals, ROS and RNS arise from a variety of sources. Internally, the primary sources of these reactive species include infiltrating immune cells-such as macrophages, eosinophils, and neutrophils—and bronchial epithelial cells that express dual oxidase 1 (Hsieh et al., 2022). Mitochondria also constitute a critical source, particularly through the generation of superoxide anions within the intracellular environment (Stowe and Camara, 2009). Externally, oxidative stress within the airways is further intensified by exposure to environmental pollutants, including airborne allergens, fine and coarse particulate matter (PM2.5 and PM10), sulfur dioxide (SO_2_), nitrogen dioxide (NO_2_), carbon monoxide (CO), cigarette smoke, and ozone (Hsieh et al., 2022). Given the complexity and multiplicity of these oxidative sources, effective antioxidant therapies must adopt a broad-spectrum approach, addressing both endogenous metabolic imbalances and exogenous environmental insults. This complexity also supports the rationale for the multi-component nature of botanical drug formulations (Figure 1).

### 3.2 Oxidative stress-mediated inflammatory mechanisms in the pathogenesis of airway mesenchymal reprogramming in asthma

**FIGURE 2:**
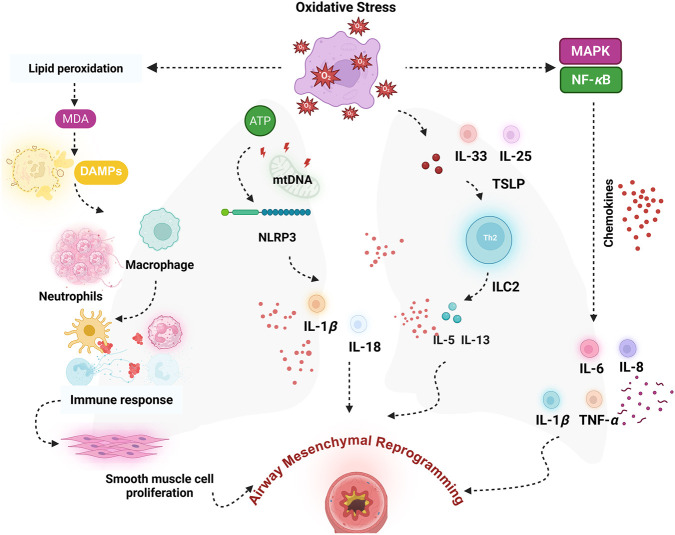
Mechanisms by which oxidative stress contributes to airway inflammation and mesenchymal reprogramming in asthma. Oxidative stress induces lipid peroxidation, leading to the generation of MDA and DAMPs, which activate neutrophils and macrophages and subsequently amplify immune responses. Mitochondrial ROS and the release of mtDNA activate the NLRP3 inflammasome, resulting in the secretion of IL-1*β* and IL-18, thereby exacerbating airway inflammation. Additionally, oxidative stress stimulates the release of epithelial-derived cytokines, including IL-25, IL-33, and TSLP, which activate ILC2s and Th2 cells, promoting the production of IL-5 and IL-13. Concurrently, the NF-*κ*B and MAPK signaling pathways are activated, facilitating the release of chemokines and pro-inflammatory cytokines (e.g., IL-6, IL-8, and TNF-*α*), further driving inflammation and airway mesenchymal reprogramming. Figure created with BioRender.com.

Oxidative stress serves as a crucial upstream mediator in asthmatic airways, initiating and intensifying immune activation and functioning as a central contributor to the persistence of chronic inflammation. When oxidative balance is disrupted, excessive ROS accumulation inflicts substantial damage on essential cellular components, such as lipids, proteins, and nucleic acids. These oxidative insults lead to lipid peroxidation, oxidation of proteins and destabilization of the genome, as evidenced by DNA damage (Juan et al., 2021). Malondialdehyde (MDA), a major byproduct formed during lipid peroxidation, is frequently utilized as a biochemical marker for evaluating oxidative burden and assessing lipid membrane injury in the context of asthma. Upon damage, cells release damage-associated molecular patterns, which are recognized by macrophages and neutrophils, thereby activating the innate immune response (Albano et al., 2022). Concurrently, ROS stimulate redox-sensitive signaling cascades, including the nuclear factor kappa B (NF-*κ*B) and mitogen-activated protein kinase (MAPK) pathways, thereby enhancing the transcription of proinflammatory cytokines such as interleukin (IL)-6, IL-8, IL-1*β*, and tumor necrosis factor-alpha (TNF-*α*), along with chemokines that recruit and activate neutrophils and eosinophils (Lee et al., 2021; Ilari et al., 2025). The release of these pro-inflammatory factors contributes to a cyclical amplification of immune activity, fostering a sustained and escalating inflammatory response within the airways (Huang et al., 2024). Simultaneously, excessive ROS impair mitochondrial structure and disrupt its functional stability, triggering the liberation of mitochondrial DNA (mtDNA) and adenosine triphosphate (ATP), both of which function as intracellular danger signals, amplifying immune responses. These danger-associated signals trigger the activation of the NOD-like receptor family pyrin domain containing 3(NLRP3) inflammasome, which in turn facilitates the processing and release of proinflammatory cytokines IL-1*β* and IL-18, thereby intensifying the proinflammatory microenvironment (Kim et al., 2014; Hu et al., 2021). Additionally, ROS stimulate airway epithelial cells to secrete key cytokines, including IL-33, IL-25, and thymic stromal lymphopoietin. These mediators play a central role in skewing immune responses toward a T helper 2 (Th2) phenotype and in initiating the activation of type 2 innate lymphoid cells, thereby promoting allergic inflammation. Acting in concert, these immune effectors promote the production of IL-5 and IL-13, which are pivotal in orchestrating type 2 immune-driven inflammation (Duchesne et al., 2022; Michaeloudes et al., 2022). The cascade of interactions between activated immune cells and structural airway cells exacerbates smooth muscle cell proliferation, ECM deposition, and epithelial cell injury and aberrant repair, sustaining the release of profibrotic mediators and ultimately promoting the occurrence and progression of asthma-associated airway mesenchymal reprogramming (Lee and Yang, 2012). Taken together, these mechanistic findings underscore the pivotal role of oxidative stress as a central mediator bridging chronic airway inflammation with airway mesenchymal reprogramming in asthma. Early and precise regulation of oxidative stress pathways may constitute a critical therapeutic approach to simultaneously curb inflammatory escalation and prevent airway structural deterioration (Figure 2).

### 3.3 Oxidative stress initiates airway EMT

**FIGURE 3:**
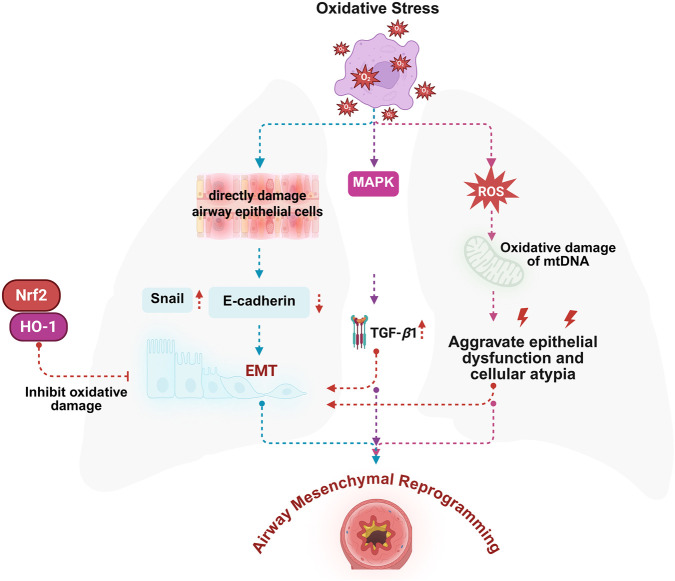
Oxidative stress-induced EMT contributes to airway mesenchymal reprogramming in asthma and is regulated by the Nrf2/HO-1 antioxidant pathway. Excessive oxidative stress is a key driving factor in airway mesenchymal reprogramming in asthma, primarily through the induction of epithelial injury and EMT. The underlying mechanisms include: 1. Oxidative stress directly damages airway epithelial cells, leading to the upregulation of EMT-related transcription factors such as Snail and the downregulation of epithelial adhesion molecules like E-cadherin, thereby initiating the EMT process; 2. Oxidative stress activates intracellular signaling cascades, such as the MAPK pathway, which promote the expression of TGF-β1, a central mediator of EMT and mesenchymal reprogramming; 3. Accumulation of ROS induces oxidative damage to mtDNA, further exacerbating epithelial dysfunction and cellular dysplasia. These events collectively contribute to the phenotypic transition of epithelial cells, aberrant cell proliferation, and ECM accumulation, ultimately resulting in airway mesenchymal reprogramming. The Nrf2/HO-1 signaling axis represents a classical antioxidant pathway that mitigates oxidative injury and suppresses EMT progression, highlighting its potential as a therapeutic target for alleviating asthma-associated airway mesenchymal reprogramming. Figure created with BioRender.com.

EMT is a fundamental pathological mechanism underlying airway mesenchymal reprogramming in asthma, and oxidative stress functions as a major trigger in initiating and sustaining this process. Under oxidative conditions, airway epithelial cells undergo structural and functional changes, including the loss of polarity and intercellular adhesion, and transition into mesenchymal-like cells with enhanced migratory capacity, invasiveness, and the ability to synthesize ECM components. This phenotypic shift facilitates structural changes such as basement membrane thickening, smooth muscle proliferation, and excessive mucus secretion (Zhang et al., 2025; Saha et al., 2022). Excessive ECM deposition increases airway stiffness and simultaneously generates mechanical signals that further promote EMT, forming a self-reinforcing and potentially difficult-to-reverse feedback loop (Varricchi et al., 2024). Oxidative stress not only serves as a direct instigator of EMT but also modulates its progression through the regulation of multiple signaling cascades. Elevated ROS levels compromise epithelial integrity and disrupt barrier function, facilitating EMT initiation (Teyani et al., 2024). ROS also induce EMT by activating transcription factors like Snail (Nikitovic et al., 2013), Concurrently suppress E-cadherin and disrupt adherens junctions, promoting epithelial susceptibility to mesenchymal reprogramming (Cano et al., 2000). Moreover, oxidative damage to mtDNA contributes to mitochondrial dysfunction, which further facilitates the phenotypic shift of epithelial cells toward a mesenchymal state (O'Rourke et al., 2002). ROS-induced activation of signaling pathways, including phosphoinositide 3-kinase/protein kinase B (PI3K/Akt) and MAPK, stimulates the release of profibrotic cytokines-notably transforming growth factor-beta 1 (TGF-*β*1)—which act in concert to accelerate EMT and enhance airway structural remodeling (Koundouros and Poulogiannis, 2018; Jiang et al., 2017). Nrf2 functions as a pivotal transcriptional regulator responsive to oxidative stress, playing a central role in the cellular antioxidant defense system. Among its downstream targets, heme oxygenase-1 (HO-1) is considered a key effector gene contributing to redox homeostasis. Acting at the core of the intracellular oxidative stress response, the Nrf2-HO-1 pathway plays a pivotal role in maintaining redox equilibrium and orchestrating antioxidant defenses (Loboda et al., 2016). Activation of this pathway facilitates the neutralization of ROS and restores redox equilibrium, thereby indirectly mitigating EMT driven by oxidative stress (Shi et al., 2022). Pharmacological enhancement of Nrf2/HO-1 activity not only reinforces antioxidant capacity but also suppresses EMT-associated aberrant remodeling, offering a novel therapeutic avenue for targeting asthma-related airway structural changes. Overall, these mechanistic insights underscore the dual role of oxidative stress in EMT regulation-functioning both as a direct effector of epithelial transformation and as a modulator through redox-sensitive signaling pathways. Intervening in the interaction of oxidative stress with structural remodeling presents a promising strategy for attenuating airway mesenchymal reprogramming and improving outcomes in asthma management (Figure 3).

### 3.4 ASM dysfunction driven by oxidative stress

**FIGURE 4:**
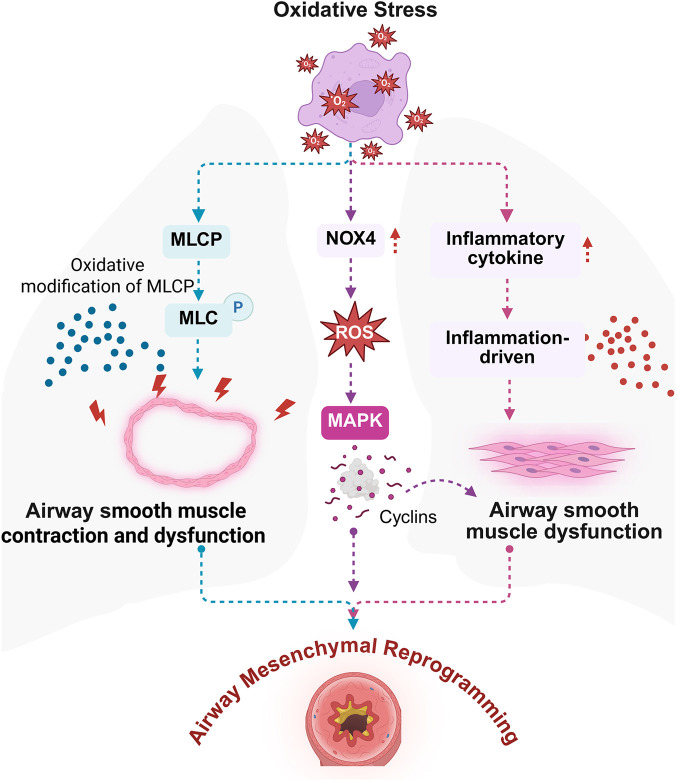
Oxidative stress exacerbates airway smooth muscle dysfunction, contributing to airway mesenchymal reprogramming in asthma. Oxidative stress activates NOX4, leading to the generation of ROS and the promotion of inflammatory responses. Inflammatory cytokines further amplify the inflammatory cascade, while activation of MAPK signaling and cyclin-dependent pathways contributes to airway smooth muscle dysfunction. Oxidative modifications of MLCP enhance smooth muscle contractility and impair normal function, ultimately resulting in airway mesenchymal reprogramming. Figure created with BioRender.com.

ASM is an essential component of the bronchial wall, and its dysfunction is critically involved in the initiation and progression of airway mesenchymal reprogramming in asthma. Aberrant ASM activity contributes directly to bronchoconstriction, airway hyperresponsiveness, and persistent structural alterations (Doeing and Solway, 2013). Among the underlying mechanisms, oxidative stress has emerged as a central factor driving both ASM hyperplasia and enhanced contractility, positioning it as a key pathological determinant of ASM dysfunction (Dai et al., 2025). The disruption of redox homeostasis in ASMCs thereby alters the regulation and function of contractile proteins. Experimental evidence indicates that ROS can induce oxidative modifications in myosin light chain phosphatase, impairing its enzymatic activity and leading to sustained phosphorylation of the myosin light chain. This prolongs ASMCs contraction and contributes to contractile dysfunction (Pan et al., 2019). In parallel, oxidative stress upregulates NADPH oxidase 4 (NOX4), an enzyme that amplifies intracellular ROS production. This ROS surge activates MAPK signaling cascades, promoting cyclin upregulation, facilitating cell cycle progression, and ultimately driving ASMCs proliferation (Dai et al., 2025). In addition, oxidative stress promotes the release of inflammatory mediators that amplify ASMCs contraction and stimulate their proliferative capacity. This sustained inflammatory and oxidative environment perpetuates ASM dysfunction and accelerates the development of structural changes in asthmatic airways (Li et al., 2022). Collectively, these insights highlight the pathological interplay between oxidative stress and ASM dysregulation, suggesting that therapeutic strategies aimed at restoring redox balance and targeting oxidative stress-induced ASM abnormalities may hold promise for attenuating airway mesenchymal reprogramming in asthmatic patients (Figure 4).

### 3.5 Oxidative stress impairs the antioxidant enzyme system in patients with asthma

**FIGURE 5:**
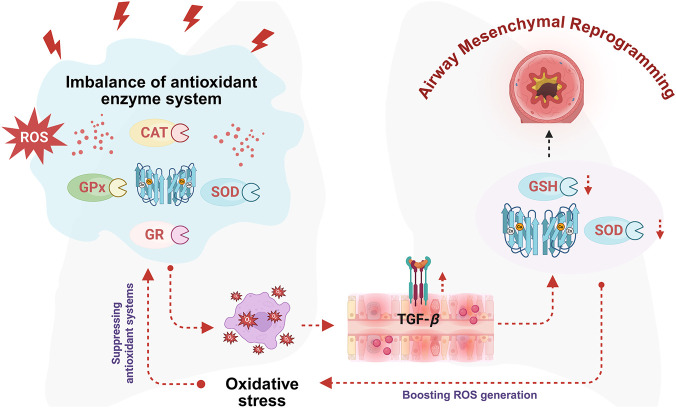
Oxidative stress impairs the antioxidant enzyme system in asthma. Excessive production of ROS stimulates epithelial cells to secrete TGF-*β*, which subsequently downregulates key antioxidants such as GSH and SOD. This reduction in antioxidant capacity exacerbates oxidative stress and intensifies redox imbalance. The resulting chronic oxidative burden contributes to sustained tissue injury and progressive airway mesenchymal reprogramming. Figure created with BioRender.com.

Asthma-associated airway mesenchymal reprogramming is commonly linked to dysfunction in the antioxidant enzyme system, leading to a disrupted equilibrium between oxidant production and antioxidant defense mechanisms, thereby promoting a persistent oxidative stress state. This enzymatic defense network is critically involved in cellular protection and comprises a series of key enzymes including superoxide dismutase (SOD), catalase (CAT), glutathione peroxidase (GPx), and glutathione reductase (GR). Collectively, these enzymes act in concert to regulate intracellular redox balance by detoxifying ROS and scavenging free radicals, thus preserving cellular integrity and preventing oxidative damage (Asatiani et al., 2025). Nevertheless, emerging evidence indicates that elevated levels of ROS can stimulate airway epithelial cells to produce TGF-*β*—a cytokine that downregulates levels of critical antioxidant components, including glutathione (GSH) and SOD. This negative regulation amplifies oxidative stress and further destabilizes redox homeostasis within cells (Hsieh et al., 2022). Prolonged exposure to such oxidative imbalances contributes to progressive tissue injury and promotes long-term airway mesenchymal reprogramming. Therefore, strategies aimed at enhancing antioxidant enzyme activity, improving airway antioxidant capacity, and restoring redox balance may represent a potential therapeutic approach for modulating asthma-associated airway mesenchymal reprogramming and limiting the progression of structural damage (Figure 5).

## 4 Key signaling pathways mediating oxidative stress-driven airway mesenchymal reprogramming in asthma

### 4.1 Nrf2/HO-1 signaling pathway

**FIGURE 6:**
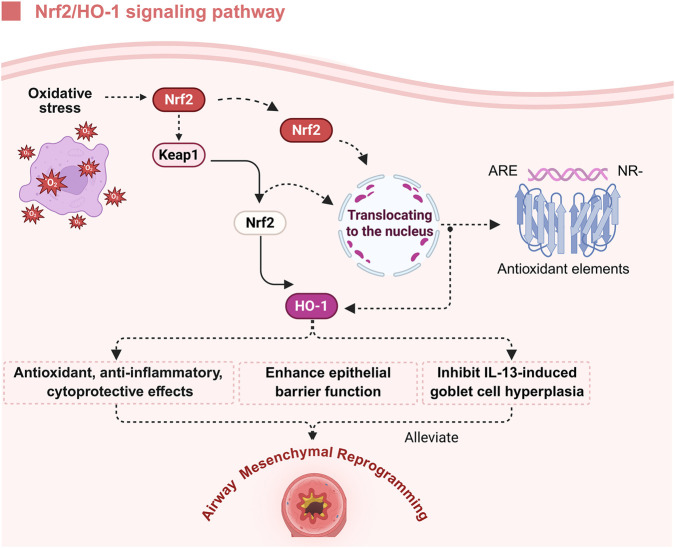
Protective role of the Nrf2/HO-1 pathway in alleviating oxidative stress and airway mesenchymal reprogramming in asthma. Following oxidative stress, Nrf2 is activated and translocates to the nucleus, where it binds to AREs to promote the transcription of antioxidant genes. Activation of Nrf2 induces the upregulation of HO-1, which exerts antioxidative, anti-inflammatory, and cytoprotective effects. Additionally, HO-1 enhances epithelial barrier integrity and suppresses IL-13-induced goblet cell hyperplasia, thereby contributing to the attenuation of airway mesenchymal reprogramming in asthma. Figure created with BioRender.com.

The Nrf2/HO-1 signaling cascade constitutes a fundamental intracellular defense system against oxidative and electrophilic insults, playing an essential role in preserving redox balance. It has demonstrated significant antioxidant and cytoprotective effects across various chronic inflammatory diseases, particularly in oxidative stress-mediated airway mesenchymal reprogramming in asthma (Boutten et al., 2011). Nrf2, classified within the CNC-bZIP transcription factor subfamily, acts as a redox sensor, responding to intracellular oxidative perturbations. Under physiological conditions, Nrf2 remains anchored in the cytoplasm through its interaction with its inhibitory partner, Kelch-like ECH-associated protein 1 (Keap1), which facilitates its continuous ubiquitination and proteasomal degradation, thereby maintaining low basal activity (Kizhner et al., 2011; Suzuki et al., 2023). Upon oxidative or electrophilic stimulation, cysteine residues on Keap1 undergo covalent modifications, inducing conformational changes that disrupt the Keap1-Nrf2 complex (Bass-Stringer et al., 2018). This dissociation stabilizes Nrf2, enabling its translocation into the nucleus, where it engages antioxidant response elements and transcriptionally activates a broad array of antioxidant, detoxifying, and cytoprotective genes (Audousset et al., 2021). Among these, HO-1 serves as a key downstream effector. HO-1 enzymatically degrades heme, yielding ferrous iron, CO, and biliverdin. Biliverdin, CO, and iron-three products of HO-1 activity-collectively contribute to redox balance and inflammation control: biliverdin neutralizes ROS, CO modulates MAPK and NF-*κ*B pathways to exert anti-inflammatory effects, and free iron is sequestered by ferritin to limit oxidative damage (Dougherty et al., 2006; Danke and Kwok, 2003). Collectively, HO-1 imparts antioxidative, anti-inflammatory, and cytoprotective functions. In the asthmatic airway, exposure of epithelial cells and macrophages to allergens, environmental pollutants, or microbial agents significantly increases ROS production, initiating oxidative stress responses (Diaz et al., 2009; Rizzi et al., 2011). In this context, rapid activation of the Nrf2/HO-1 axis occurs, resulting in robust induction of HO-1 and the establishment of an endogenous defense mechanism to counteract oxidative injury and modulate inflammation (Ge et al., 2024). Empirical evidence suggests that Nrf2/HO-1 activation effectively reduces excessive ROS, attenuates inflammatory cytokine production, inhibits aberrant ASMCs proliferation, and alleviates airway hyperresponsiveness-associated structural remodeling (Saunders et al., 2022). Additionally, activation of this pathway promotes the induction of epithelial tight junction proteins such as E-cadherin and zonula occludens-1 (ZO-1), thereby enhancing barrier integrity, reducing antigen translocation, and limiting secondary inflammatory cascades (Sussan et al., 2015). Importantly, elevated HO-1 expression has been shown to inhibit interleukin-13 (IL-13)-driven goblet cell hyperplasia, thus reducing mucus overproduction and relieving mucus-associated airway obstruction and airflow limitation (Geng et al., 2024). In summary, the Nrf2/HO-1 signaling pathway exerts crucial protective effects in multiple pathological processes of asthma-associated airway mesenchymal reprogramming by coordinating the regulation of oxidative stress, inflammatory responses, epithelial barrier function, and mucus secretion. These findings provide a solid theoretical basis and identify potential molecular targets for therapeutic strategies aimed at modulating oxidative stress-related mechanisms in asthma (Figure 6).

### 4.2 NF-*κ*B signaling pathway

**FIGURE 7:**
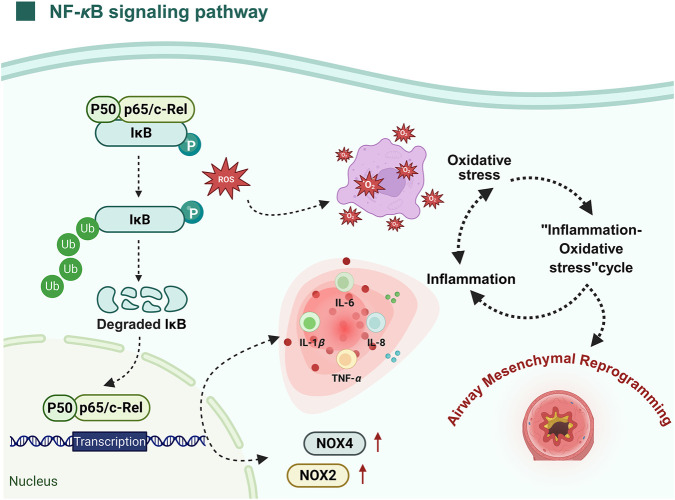
Role of oxidative stress in activating the NF-*κ*B signaling pathway and its impact on airway mesenchymal reprogramming in asthma. Following oxidative stress, ROS activate the NF-*κ*B complex by inducing the phosphorylation and degradation of I*κ*B. This degradation releases the NF-*κ*B dimers (p50 and p65/c-Rel), allowing their translocation into the nucleus, where they initiate the transcription of pro-inflammatory cytokines such as TNF-*α*, IL-6, IL-8, and IL-1*β*. These cytokines further amplify inflammation, creating a self-perpetuating feedback loop of oxidative stress. Moreover, NF-*κ*B activation promotes the upregulation of NOX2 and NOX4, which enhances ROS production and aggravates oxidative stress. The persistent cycle of inflammation and oxidative stress plays a pivotal role in the pathogenesis of airway mesenchymal reprogramming in asthma. Figure created with BioRender.com.

As a central transcriptional regulator, the NF-*κ*B pathway governs the coordination of innate and adaptive immune mechanisms. Its sustained activation has been closely linked to the development and progression of various chronic inflammatory conditions, particularly in driving ongoing airway inflammation and contributing to structural alterations observed in asthma (Mishra et al., 2018). Accumulating research highlights oxidative stress as an important initiator of NF-*κ*B pathway activation, positioning it as a key modulator in inflammation-related signaling cascades. Elevated ROS stimulate the activation of the I*κ*B kinase (IKK) complex, resulting in the ubiquitin-dependent phosphorylation and subsequent degradation of the inhibitory molecule inhibitor of kappa B alpha (I*κ*B*α*) via the proteasome system. This degradation liberates the NF-*κ*B heterodimer, primarily consisting of p65 and p50 subunits, from its cytoplasmic anchor, allowing its entry into the nucleus (Pantano et al., 2008). Upon nuclear entry, NF-*κ*B interacts with specific promoter regions on DNA, initiating the transcription of numerous pro-inflammatory mediators. These include key cytokines such as TNF-*α*, IL-1*β*, IL-6, and IL-8, along with various chemokines, adhesion proteins, and regulatory growth factors. These mediators further promote immune cell recruitment, adhesion, and activation, perpetuating a chronic inflammatory microenvironment (Barnes and Karin, 1997). Notably, oxidative stress is also modulated by the upregulation of subunits of the nicotinamide adenine dinucleotide phosphate (NADPH) oxidase enzyme system by NF-*κ*B-particularly NOX2 and NOX4 subunits-which, in turn, elevate intracellular ROS levels. This bidirectional interaction creates a self-amplifying loop wherein oxidative stress and inflammation potentiate one another, exacerbating airway pathology (Mittal et al., 2014). Crucially, sustained activation of NF-*κ*B extends beyond inflammation, directly contributing to structural alterations within the airway. These include impaired epithelial regeneration, increased proliferation and hypertrophy of ASMCs, goblet cell metaplasia, and dysregulated deposition of ECM proteins. Collectively, these pathological changes result in airway wall thickening, luminal narrowing, and compromised pulmonary function (Sahiner et al., 2011; Burton and Jauniaux, 2011). In summary, the NF-*κ*B signaling cascade functions as a central molecular nexus linking oxidative stress to chronic airway inflammation and remodeling, underscoring its essential role in asthma pathogenesis. Therapeutic strategies targeting NF-*κ*B and its upstream ROS signals may represent a potential approach for modulating chronic airway inflammation and limiting inflammation-associated structural alterations in asthma (Figure 7).

### 4.3 MAPK signaling pathway

**FIGURE 8:**
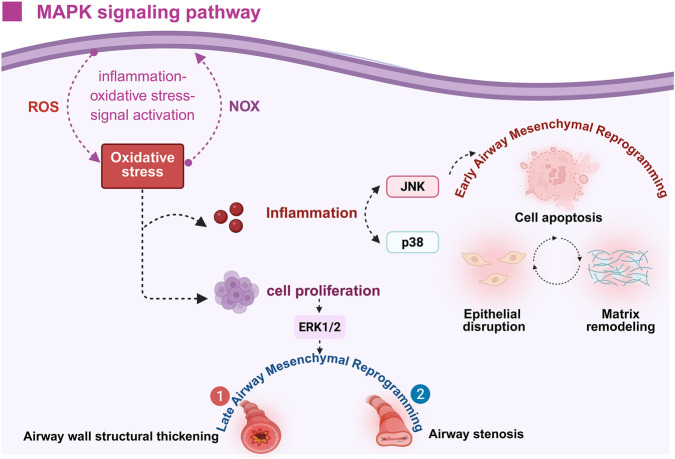
Role of oxidative stress in activating the MAPK signaling pathway and its impact on early and late stages of airway mesenchymal reprogramming in asthma. Oxidative stress induced by ROS and NOX activates the MAPK pathway, contributing to two distinct phases of airway mesenchymal reprogramming. In the early stage, activation of JNK and p38 MAPK promotes inflammation, leading to epithelial cell damage, extracellular matrix remodeling, and apoptosis. In the late stage, activation of ERK1/2 drives cellular proliferation, resulting in thickening of the airway wall and airway narrowing. Figure created with BioRender.com.

The MAPK pathway constitutes a broadly conserved intracellular signaling network in eukaryotes, acting as a pivotal hub that transduces diverse external signals—such as ROS, mitogenic stimuli, and inflammatory mediators—into appropriate cellular responses (Braicu et al., 2019). This signaling network consists of three major branches: extracellular signal-regulated kinases 1 and 2 (ERK1/2), c-Jun N-terminal kinase (JNK), and p38 MAPK, each governing specific biological processes such as cell proliferation, differentiation, immune activation, and programmed cell death. Collectively, these cascades play a role in modulating airway inflammation and structural remodeling in the context of asthma (Burton and Jauniaux, 2011). Among the MAPK subfamilies, JNK and p38 MAPK exhibit heightened sensitivity to oxidative insults. Upon activation, these kinases promote the transcription of pro-inflammatory mediators and apoptosis-related genes, resulting in epithelial cell death, degradation of tight junction proteins, and compromised barrier integrity. These changes contribute to early structural alterations, including epithelial injury and ECM remodeling (Soga et al., 2012). In contrast, the ERK1/2 branch primarily regulates cell survival and proliferation. In ASMCs, sustained oxidative stress-induced activation of ERK1/2 promotes abnormal cellular proliferation and hypertrophy, thereby contributing to airway wall thickening and luminal narrowing, which are hallmark features of advanced airway remodeling (Burton and Jauniaux, 2011). Importantly, a bidirectional regulatory relationship exists between the MAPK pathway and oxidative stress. On one hand, ROS and RNS can activate upstream MAPK kinases through oxidative modifications, initiating the MAPK signaling cascade (Rahman, 2002). On the other hand, sustained stimulation of the MAPK pathway amplifies both the expression and catalytic function of key ROS-producing enzymes, notably NADPH oxidase (NOX) isoforms. This process further elevates local oxidative burden, establishing a self-reinforcing loop of inflammation, oxidative stress, and MAPK pathway activation (Pandey and Fulton, 2011; Pelaia et al., 2021). In summary, the MAPK signaling network serves as a pivotal molecular intermediary linking oxidative stress to diverse cellular dysfunctions. This mechanism is deeply implicated in asthma development, as it intensifies immune-driven inflammation, compromises the integrity of the epithelial barrier, and contributes to both the expansion and thickening of ASM tissue. As such, MAPK signaling represents an important mechanistic conduit through which oxidative stress drives the progression of airway mesenchymal reprogramming (Figure 8).

### 4.4 PI3K/Akt signaling pathway

**FIGURE 9:**
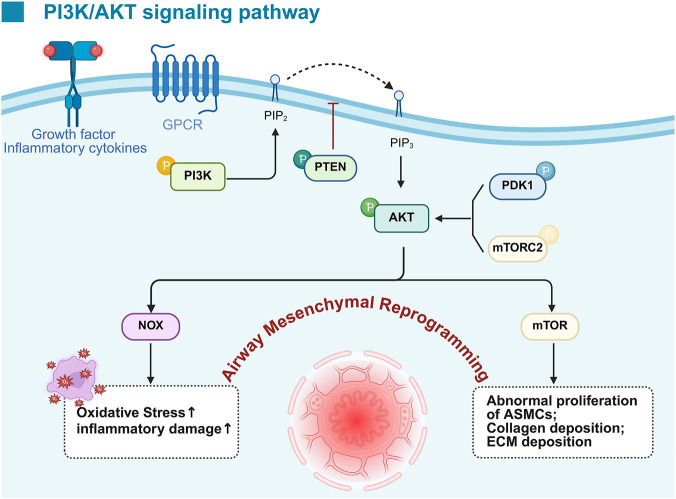
The PI3K/AKT signaling pathway synergistically enhances the role of oxidative stress in airway mesenchymal reprogramming in asthma. Growth factors and pro-inflammatory cytokines activate upstream membrane receptors, leading to PI3K activation and conversion of PIP2 into PIP3. This process is negatively regulated by PTEN. Accumulation of PIP3 promotes the recruitment and activation of AKT, which is phosphorylated by PDK1 and mTORC2. Activation of this pathway promotes NOX-mediated increases in oxidative stress and inflammatory injury, thereby contributing to airway mesenchymal reprogramming. Additionally, mTOR activation drives abnormal proliferation of ASMCs, collagen deposition, and ECM accumulation. Figure created with BioRender.com.

The pathway, known as the PI3K/Akt cascade, serves as a key intracellular signaling mechanism that mediates cellular responses to environmental stimuli. It plays an essential role in orchestrating various biological and pathological events, including proliferation, differentiation, cell survival, and metabolic homeostasis (Ghafouri-Fard et al., 2021). Activation of this signaling cascade is generally initiated when extracellular ligands—such as growth factors and inflammatory cytokines—bind to membrane-bound receptors, including receptor tyrosine kinases and G protein-coupled receptors (Hennessy et al., 2005). In asthma pathogenesis, class I PI3K isoforms, particularly the p110δ and p110γ catalytic subunits that are predominantly expressed in immune cells, play critical roles in intensifying inflammatory signaling and promoting structural changes within the airway (Camps et al., 2005). Enhanced PI3K/Akt pathway signaling has been identified in airway epithelial cells as well as ASMCs among individuals with asthma, suggesting its ongoing involvement in disease pathology (O'Sullivan et al., 2021; Wang et al., 2014; Krymskaya et al., 2001). Following its activation, PI3K facilitates the conversion of phosphatidylinositol 4,5-bisphosphate into phosphatidylinositol 3,4,5-trisphosphate, a lipid-derived secondary messenger that mediates the membrane localization of Akt. To achieve full activation, Akt must undergo sequential phosphorylation events—first at threonine 308 by phosphoinositide-dependent kinase-1, followed by phosphorylation at serine 473 mediated by the mechanistic target of rapamycin complex 2 (Manning and Toker, 2017). Activated Akt contributes to disease pathology through multiple downstream mechanisms that promote oxidative stress and inflammation. One primary effect of Akt activation is the upregulation of NOX expression and function, which elevates ROS generation and aggravates both oxidative damage and pro-inflammatory signaling cascades (Nakanishi et al., 2014). Secondly, Akt modulates the mTOR pathway, which drives abnormal ASMCs proliferation and stimulates excessive collagen synthesis and ECM deposition—processes that underlie airway wall thickening and structural remodeling (Song et al., 2025). Of particular significance, phosphatase and tensin homolog—a well-established tumor suppressor—acts as the primary inhibitory modulator of the PI3K/Akt signaling cascade, counterbalancing its activation to maintain cellular homeostasis. This reduction in inhibitory control allows for sustained PI3K/Akt activation, thereby reinforcing chronic inflammation and promoting progressive airway remodeling (Nakanishi et al., 2014). In summary, by orchestrating redox imbalance, sustaining inflammatory cascades, and driving pathological structural changes within the airway, the PI3K/Akt signaling pathway serves as a central molecular hub in asthma-associated airway mesenchymal reprogramming, highlighting its potential relevance as a therapeutic target for intervention (Figure 9).

## 5 Metabolites originating from botanical drugs

Botanical drugs used in TCM provide a chemically diverse source of small molecules relevant to asthma management. These metabolites may counter oxidative stress by reinforcing endogenous antioxidant defenses and, in parallel, modulate key features of airway mesenchymal reprogramming, including inflammation, epithelial plasticity/EMT, and ASMC proliferation/phenotypic remodeling (Xu et al., 2024; Meng et al., 2022). A growing body of experimental studies suggests that selected botanical-derived metabolites can influence processes related to airway mesenchymal reprogramming in asthma (Wang et al., 2019; Han et al., 2022; Peng et al., 2024). However, the available evidence is derived mainly from preclinical studies and remains subject to certain limitations.

### 5.1 Flavonoids

Flavonoids are plant-derived secondary metabolites that have been widely studied for redox- and inflammation-modulating activities in asthma-relevant models (Qin et al., 2024a; Geng et al., 2024; Pyo et al., 2024). Among the extensively studied flavonoids, quercetin is a tetrahydroxyflavone commonly found in food-medicinal plants such as *Malus domestica* Borkh. (apple) (Khan et al., 2013). Quercetin enhances pulmonary antioxidant capacity by upregulating GSH and downregulating MDA, a key indicator of lipid peroxidation. At the same time, it downregulates pro-inflammatory factors, including cyclooxygenase-2 (COX-2), TNF-*α*, IL-4, IL-6, and IL-8, and directly suppresses the proliferation of ASMCs, thereby contributing to the attenuation of structural remodeling (Hsieh et al., 2022; Liu et al., 2024; Peng et al., 2018). Within a mesenchymal reprogramming framework, the most relevant link is the reported suppression of ASMC proliferation, which may limit airway wall thickening and downstream remodeling phenotypes. Derived from the dried root of *Scutellaria baicalensis* Georgi, baicalin—a flavonoid glycoside—has demonstrated pharmacological activity in preclinical models of asthma (Zhang et al., 2011). Preclinical evidence suggests that baicalin may attenuate epithelial plasticity by upregulating the epithelial marker E-cadherin and simultaneously suppressing mesenchymal indicators like N-cadherin and alpha-smooth muscle actin (*α*-SMA), consistent with inhibition of EMT under oxidative-stress conditions, which represents an important component of airway mesenchymal reprogramming. This regulatory effect has been reported to attenuate ROS-induced EMT and mitigate oxidative cellular damage (Zhai and Wang, 2022; Li et al., 2024a). In addition, baicalin inhibits the proliferation of ASMCs and reduces the levels of Th2-associated cytokines, such as IL-4 and IL-13, thereby helping preserve airway architecture and limiting fibrotic tissue remodeling (Sun et al., 2013; Mabalirajan et al., 2013; Hu et al., 2023). Because EMT reflects epithelial-to-mesenchymal plasticity and acquisition of mesenchymal features (e.g., *α*-SMA), baicalin’s marker-level effects provide mechanistic support for its involvement in airway mesenchymal reprogramming. Galangin, a trihydroxyflavone present in medicinal and culinary plants like *Alpinia officinarum* Hance and *Curcuma longa* L., has also shown protective effects against airway remodeling. Galangin exerts its effects by limiting ROS production triggered by TGF-*β*1 stimulation, while simultaneously blocking phosphorylation events associated with the activation of Akt and MAPK signaling cascades (Wang et al., 2023a). These actions collectively reduce inflammatory cell infiltration and inhibit ASMC proliferation, thereby attenuating pathological structural changes in the airway wall (Liu et al., 2015). Within a mesenchymal reprogramming framework, inhibition of the TGF-*β*1–ROS–Akt/MAPK axis links oxidative-stress signaling to reduced structural-cell activation and ASMC expansion, consistent with mitigation of remodeling-related phenotypes. Taken together, flavonoids may modulate asthma-associated airway mesenchymal reprogramming through convergent effects on epithelial plasticity/EMT and ASMC remodeling under oxidative-stress. However, flavonoids have complex sources and variable exposure, so attributing effects to individual compounds should be interpreted cautiously.

### 5.2 Terpenoids

Terpenoids are a class of plant-derived secondary metabolites biosynthesized from isoprene building blocks and display diverse carbon scaffolds. Given their redox- and inflammation-modulating properties, terpenoids have been explored for their ability to influence reprogramming-relevant structural-cell phenotypes in asthma, including epithelial plasticity/EMT and ASMC expansion/phenotypic remodeling (Liao et al., 2023; Juergens, 2014). Among the well-characterized terpenoids, triptolide, a diterpenoid lactone extracted from *Tripterygium wilfordii* Hook. f., has been reported to show anti-remodeling activity. It suppresses ASMC proliferation, downregulates key inflammatory signaling pathways, and inhibits pro-inflammatory mediator production, effects that are consistent with limiting airway wall thickening and downstream remodeling (Yuan et al., 2019; Chen et al., 2015; He et al., 2020; Chen et al., 2014). Another compound, andrographolide, isolated from *Andrographis paniculata*, has shown efficacy in ovalbumin (OVA)-sensitized murine asthma models. By suppressing NF-*κ*B signaling and preventing the assembly of the NLRP3 inflammasome—in association with reduced intracellular ROS—it has been reported to reduce the concentrations of IL-6, IL-17A, IL-17F, TNF-*α*, and IL-1*β* (Yu et al., 2021; Peng et al., 2016). Furthermore, in epithelial cell models, andrographolide inhibits TGF-*β*1-induced ROS production and attenuates EMT-associated marker changes, supporting a mechanistic link between redox signaling and epithelial plasticity within a mesenchymal reprogramming framework (Yu et al., 2021; Peng et al., 2016; Li et al., 2020). Artesunate, a semi-synthetic sesquiterpene lactone derived from the antimalarial agent artemisinin of *Artemisia annua* L., has also been reported to show anti-remodeling properties. In murine models of asthma, artesunate suppresses the expression of inducible nitric oxide synthase (iNOS) and NOX isoforms, while markedly lowering oxidative stress indicators, including 8-iso-prostaglandin F_2_α (8-iso-PGF_2_α), 8-hydroxy-2′-deoxyguanosine (8-OHdG), and 3-nitrotyrosine. Simultaneously, it triggers the Nrf2/HO-1 signaling cascade, suppresses ASMC proliferation, and mitigates inflammatory cell infiltration, collectively alleviating airway structural remodeling (Ho et al., 2012). Collectively, terpenoids are most often linked to NF-*κ*B/NLRP3- and Nrf2/HO-1–associated redox control that may modulate airway mesenchymal reprogramming, although the evidence base remains limited and heterogeneous.

### 5.3 Coumarins

Coumarins, a class of plant-derived secondary metabolites originating from botanical drugs, are structurally characterized by a benzopyran-2-one (benzo-α-pyrone) scaffold. In the context of asthma-related airway mesenchymal reprogramming, current evidence more consistently links coumarins to remodeling-relevant structural outcomes, including subepithelial fibrosis/ECM remodeling and goblet-cell hyperplasia, often accompanied by changes in antioxidant capacity or cytokine-related responses. Esculetin, a hydroxylated derivative of coumarin obtained from Cortex fraxini, has shown notable efficacy in OVA-induced asthma models. Its benefits include reducing airway hyperresponsiveness, inhibiting the release of Th2-associated cytokines, and decreasing eosinophil accumulation within bronchoalveolar lavage fluid (BALF). Beyond inflammation control, esculetin has been associated with reduced 15-lipoxygenase (15-LOX) activity, decreased lipid peroxidation, and restoration of mitochondrial cytochrome c oxidase function. Concomitantly, TGF-*β*1 downregulation has been reported alongside attenuation of subepithelial fibrosis, supporting a mechanistic link to fibrotic/ECM-related remodeling (Mabalirajan et al., 2009). Imperatorin, a naturally occurring furanocoumarin present in Angelica dahurica, has also been reported to alleviate remodeling-relevant airway changes in asthma models. Mouse models of asthma show that imperatorin markedly decreases the infiltration of inflammatory cells and the concentration of pro-inflammatory cytokines in BALF. At the same time, it mitigates airway inflammation, restrains goblet-cell hyperplasia, and reduces ECM accumulation. Its beneficial effects are partly attributed to stimulation of the Nrf2/HO-1 signaling pathway, which boosts the activity of pivotal antioxidant enzymes, including SOD and GPx, while simultaneously lowering MDA concentrations. Moreover, imperatorin interferes with several signaling cascades implicated in the development of asthma, notably PI3K/Akt, MAPK, and NF-*κ*B pathways, with concomitant decreases in remodeling-associated readouts (e.g., VEGF and *α*-SMA) and ROS (Wang et al., 2023b; Xian et al., 2020). Collectively, coumarins appear to couple redox/lipid-peroxidation control (15-LOX–linked oxidative injury and Nrf2/HO-1 signaling) to downstream structural remodeling phenotypes (subepithelial fibrosis/ECM deposition and goblet-cell hyperplasia) relevant to airway mesenchymal reprogramming; however, the evidence base remains limited.

### 5.4 Glycosides

Glycosides often exist as glycosylated conjugates of aglycones and can undergo enzymatic hydrolysis to release the active aglycone. Representative metabolites include rosavins from *Rhodiola rosea* (rhodiola), which have been reported to exert a multi-pronged profile, significantly lowering levels of immunoglobulin E (IgE) and Th2 cytokines (IL-4, IL-5, IL-13), reducing ROS/MDA accumulation, and triggering the Nrf2/HO-1 pathway to boost SOD1 expression. Non-targeted metabolomics further suggests an associated shift in pulmonary metabolic profiles (Ming et al., 2022; Shan et al., 2022; Wang et al., 2023c). Polydatin, a plant-derived glycoside, has been investigated for antioxidative and anti-inflammatory activities, with reported suppression of intracellular ROS and activation of Nrf2-dependent signaling. As a result, antioxidant enzymes such as HO-1 and NADPH quinone dehydrogenase 1 (NQO1) are transcriptionally induced, thereby supporting redox homeostasis (Ye et al., 2022). In asthma models, polydatin has been reported to suppress EMT and mitigate airway inflammation by reducing levels of pro-inflammatory mediators, including IL-4, IL-5, IL-13, and TNF-*α*, consistent with modulation of oxidative stress–linked pathways relevant to airway structural alteration (Zeng et al., 2019, Hanna et al., 2019). Moreover, syringin, a bioactive phenylpropanoid glycoside obtained from *Eleutherococcus senticosus* (Rupr. and Maxim.) Maxim., has been reported to counteract oxidative lung injury through suppression of the NF-*κ*B signaling pathway. This effect manifests as decreased concentrations of MDA and myeloperoxidase (MPO) in lung tissue and BALF, accompanied by enhanced activities of SOD and GSH (Dai et al., 2021). Overall, these glycosides converge on Nrf2/HO-1– and NF-*κ*B–linked redox control with accompanying Th2-inflammatory modulation, yet their translation to clinical settings remains constrained by limited clinical validation and exposure-relevant pharmacokinetic and target-site exposure evidence.

### 5.5 Alkaloids

**FIGURE 10:**
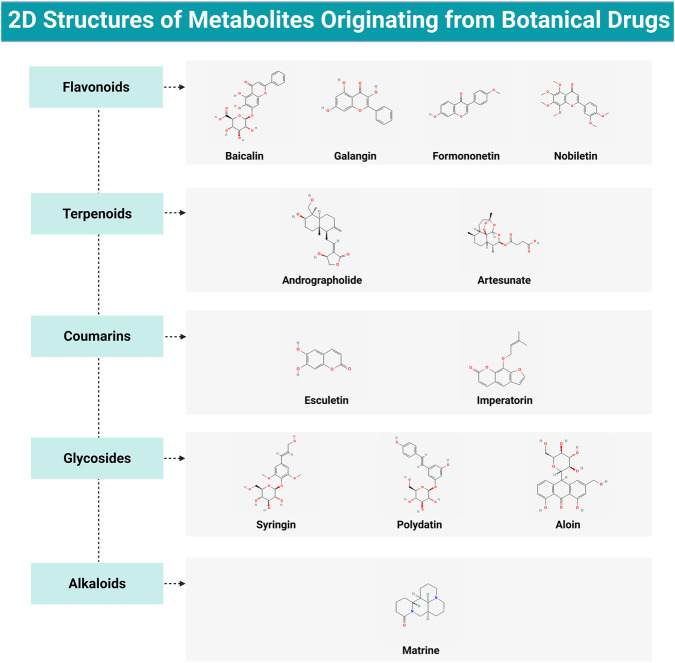
2D structures of metabolites originating from botanical drugs. Figure created with BioRender.com.

Alkaloids are nitrogen-bearing organic molecules widely distributed across the plant kingdom and have been investigated for their ability to modulate pathways relevant to asthma-associated airway mesenchymal reprogramming. Among representative alkaloids, matrine—a four-ring quinolizidine alkaloid primarily extracted from the roots of *Sophora flavescens* Aiton—has shown notable therapeutic benefits in preclinical models of asthma (You et al., 2020). In OVA-sensitized mice, matrine attenuates reprogramming-relevant airway changes by limiting inflammatory cell infiltration and excessive mucus production. This effect is accompanied by lowered concentrations of Th2 cytokines in both BALF and mediastinal lymph nodes, as well as reduced serum IgE levels. The beneficial actions of matrine have been attributed to several complementary mechanisms. First, matrine blocks the NLRP3 inflammasome–Caspase-1–IL-1*β* pathway, which consequently restrains downstream inflammatory signaling events. Second, it helps restore redox balance by boosting the activity of antioxidant enzymes, including SOD and CAT, while simultaneously reducing the buildup of MDA and ROS. Third, matrine modulates mitochondrial homeostasis via AMPK phosphorylation, which inhibits DRP1-driven mitochondrial fission and promotes mitochondrial fusion and functional integrity (Ren et al., 2024). Additionally, matrine reduces ASMC proliferation and downregulates TGF-*β*1 expression, linking redox/inflammatory control to structural-cell remodeling phenotypes relevant to airway mesenchymal reprogramming (Lei et al., 2009). However, alkaloids are diverse and target multiple pathways, which may, to some extent, limit their translational feasibility (Figure 10; Table 1).

**TABLE 1:** The mechanisms of metabolites originating from botanical drugs in modulating oxidative stress–driven airway mesenchymal reprogramming in asthma.

Metabolites	Molecular formula	Classification	Main resources	Drug name	Test models	
					*In vivo*	*In vitro*
Baicalin	C_21_H_18_O_11_	Flavonoids	*Scutellaria baicalensis* Georgi (Lamiaceae)	Scutellariae Radix (Huangqin)	OVA-induced C57BL/6 mice	PDGF-BB-induced SMCs
Galangin	C_15_H_10_O_5_	Flavonoids	*Alpinia officinarum* Hance (Zingiberaceae)	Alpiniae Officinarum Rhizoma (Gaoliangjiang)	OVA-induced BALB/c mice	TGF-*β*1-induced human ASMCs
Nobiletin	C_21_H_22_O_8_	Flavonoids	*Citrus reticulata* Blanco (Rutaceae)	Citri Reticulatae Pericarpium (Chenpi)	OVA + PM2.5-induced BALB/c mice	
Formononetin	C_16_H_12_O_4_	Flavonoids	*Trifolium pratense* L. (Fabaceae)	Trifolium pratense (Red clover)	OVA-induced BALB/c mice	
Andrographolide	C_20_H_30_O_5_	Terpenoids	*Andrographis paniculata* (Burm.f.) Wall. ex Nees (Acanthaceae)	Andrographidis Herba (Chuanxinlian)	OVA-induced C57BL/6 mice	LPS-induced RAW264.7 cells; LPS/OVA-induced BMDMs
Artesunate	C_19_H_28_O_8_	Terpenoids	*Artemisia annua* L. (Asteraceae)	NA (semi-synthetic; Qinghao)	OVA-induced BALB/c mice	TNF-*α*-induced BEAS-2B cells
Esculetin	C_9_H_6_O_4_	Coumarins	*Fraxinus chinensis subsp. rhynchophylla* (Hance) A.E.Murray (Oleaceae)	Fraxini Cortex (Qinpi)	OVA-induced BALB/c mice	
Imperatorin	C_16_H_14_O_4_	Coumarins	*Angelica dahurica* (Hoffm.) Benth. and Hook.f. ex Franch. and Sav. (Apiaceae)	Angelicae Dahuricae Radix (Baizhi)	OVA-induced BALB/c mice	OVA-induced ASMCs
Syringin	C_17_H_24_O_9_	Glycosides	*Eleutherococcus senticosus* (Rupr. and Maxim.) Maxim. (Araliaceae)	Eleutherococci Senticosi Radix et Rhizoma (Ciwujia)	OVA-induced BALB/c mice	
Polydatin	C_20_H_22_O_8_	Glycosides	*Reynoutria japonica* Houtt. (Polygonaceae)	Polygoni Cuspidati Rhizoma (Huzhang)	OVA-induced BALB/c mice	TGF-*β*1-induced BEAS-2B cells
Aloin	C_21_H_22_O_9_	Glycosides	*Aloe vera* (L.) Burm.f. (Asphodelaceae)	Aloe (Luhui)	OVA-induced C57BL/6 mice	
Matrine	C_15_H_24_N_2_O	Alkaloids	*Sophora flavescens* Aiton (Fabaceae)	Sophorae Flavescentis Radix (Kushen)	OVA-induced BALB/c mice	

Dose	Controls used	Experimental duration	Regulate oxidative stress markers	Associated signaling pathways	Affected processes of airway mesenchymal reprogramming	References
*In vivo*: Oral gavage at 50 μg/g; *In vitro*: 0.1 mg/mL	Normal control; OVA-induced model control	*In vivo*: 19 days; *In vitro*: 24 h	MDA↓,NO↓,SOD↑	TLR4/NF-*κ*B (−)	Airway inflammation, collagen deposition, and smooth muscle cell proliferation	Zhai and Wang (2022)
*In vivo*: Intraperitoneal injection at low dose 0.1 mg/kg and high dose 0.5 mg/kg; *In vitro*: 10 μM	Normal control; OVA-induced model control; Dexamethasone (positive control); Vehicle control	*In vivo*: 8 weeks; *In vitro*: 24-72 h	ROS↓,NOX4↓, SOD↑,CAT↑	TGF-*β*1-ROS-MAPK/Akt (−)	Airway inflammation, collagen deposition, smooth muscle cell proliferation, and goblet cell hyperplasia	Liu et al. (2015)
Oral gavage administration: low dose 1.8 mg/kg, medium dose 3.6 mg/kg, high dose 7.2 mg/kg	Normal control; PM2.5 model control; OVA + PM2.5-induced model control; Dexamethasone (positive control)	45 days	MDA↓,ROS↓,Keap-1↓, SOD↑,GPx↑,Nrf2↑	NF-*κ*B (−); Nrf2(+)	Airway inflammation, antioxidant enzyme system (no EMT/ASMC/ECM endpoints)	Chen et al. (2025)
Oral gavage administration: low dose 10 mg/kg, medium dose 20 mg/kg, high dose 40 mg/kg	Normal control; OVA-induced model control; Dexamethasone (positive control)	48 days	ROS↓,SOD↑,HO-1↑,SOD1↑	NF-*κ*B (−); JNK (−); Nrf2/HO-1 (+)	Airway inflammation, collagen deposition, goblet cell hyperplasia and mucus secretion, airway hyperresponsiveness	Yi et al. (2020)
*In vivo*: Intraperitoneal injection at low dose 5 mg/kg and high dose 10 mg/kg; *In vitro*: RAW264.7 cells at 1–30 μM, BMDM cells at 3–30 μM	Normal control; OVA-induced model control	*In vivo*: 28 days; *In vitro*: 7-8 days (BMDMs differentiation) + 6-24 h (stimulation)	ROS↓	NF-*κ*B (−); NLRP3 (−)	Airway inflammation; goblet-cell hyperplasia; mucus hypersecretion	Peng et al. (2016)
*In vivo*: Intraperitoneal injection at 30 mg/kg; *In vitro*: 30 μM	Normal control; OVA-induced model control; Dexamethasone (positive control); Vehicle control	*In vivo*: Not explicitly specified; *In vitro*: 2 h + 30 min	8-isoprostane↓,8-OhdG↓,3-nitrotyrosine↓,iNOS↓, SOD↑,CAT↑	Nrf2(+)	Airway inflammation; oxidative damage; antioxidant enzyme system	Ho et al. (2012)
Oral gavage administration: 1 mg/kg	Normal control; OVA-induced model control; Vehicle control	32 days	15-LOX↓,L-OOH↓,8-isoprostane↓	15-LOX (−)	Collagen deposition, goblet cell hyperplasia, mucus secretion	Mabalirajan et al. (2009)
*In vivo*: Low dose 15 mg/kg, medium dose 30 mg/kg, high dose 60 mg/kg; *In vitro*: 1 μM, 5 μM, 10 µM	Normal control; OVA-induced model control; Vehicle control	*In vivo*: 8 weeks; *In vitro*: 48 h	ROS↓	Nrf2/HO-1 (+); NF-*κ*B (−); MAPK (−); PI3K/Akt (−)	Smooth muscle cell proliferation, mucus secretion, collagen deposition, goblet cell hyperplasia	Xian et al. (2020)
Intraperitoneal injection: low dose 25 mg/kg, medium dose 50 mg/kg, high dose 100 mg/kg	Normal control; OVA-induced model control; Dexamethasone (positive control)	12 weeks	MDA↓,MPO↓,NO↓,SOD↑,GSH↑	NF-*κ*B (−)	Airway inflammation, collagen deposition, smooth muscle cell proliferation	Dai et al. (2021)
*In vivo* (method not specified): 100 mg/kg; *In vitro*: 100 μM	Normal control; OVA-induced model control	*In vivo*: 28 days; *In vitro*: 48 h	ROS↓,NOX1↓,NOX4↓, NQO1↑,HO–1↑	Nrf2/HO-1 (+)	EMT and collagen deposition	Zeng et al. (2019)
Oral gavage administration: 20 mg/kg	Normal control; OVA-induced model control; Aloin alone group	31 days	MDA↓,SOD↑,GSH↑,Nrf2↑,HO–1↑	Nrf2/HO-1 (+); TGF-*β*/Smad2/3 (−)	Airway inflammation, airway hyperresponsiveness, collagen deposition	Wei et al. (2023)
Oral gavage administration: low dose 50 mg/kg, high dose 100 mg/kg	Normal control; OVA-induced model control; Dexamethasone (positive control)	26 days	ROS↓,MDA↓,SOD↑,CAT↑	AMPK (+); DRP1 (−); NLRP3 (−); Nrf2/HO-1 (+)	Airway inflammation, mucus secretion	Ren et al. (2024)

Metabolites from botanical drugs implicated in oxidative stress–driven airway mesenchymal reprogramming in asthma. Symbols: ↑ increase; ↓ decrease; (+) activation; (−) inhibition.

## 6 Botanical drug formulations

Traditional Chinese botanical drug formulations, as multi-herb and multi-constituent interventions, have been investigated for their potential roles in asthma. By combining multiple medicinal herbs, these formulations may jointly modulate oxidative stress–related pathways and the structural-cell remodeling processes they drive.

### 6.1 Shegan mahuang decoction (SGMHD)

SGMHD is a time-honored formulation in TCM, commonly prescribed for the management of respiratory conditions characterized by cold-like manifestations, such as asthma. High-performance liquid chromatography coupled with ultraviolet detection (HPLC-UV) has identified guanosine, chlorogenic acid and tectoridin as the principal bioactive metabolites in the aqueous extract of SGMHD (Yen et al., 2014). Among these, tectoridin has been reported to enhance antioxidant defenses in BALF (increased CAT and SOD) while reducing oxidative-damage readouts (ROS and MDA), with activation of the Keap1–Nrf2–HO-1 pathway implicated (Jiang et al., 2024). In OVA-induced asthma mouse models, SGMHD administration attenuates lung pathology and improves remodeling-relevant outcomes, including reduced peribronchial/perivascular inflammatory infiltration, decreased alveolar wall thickening, less mucus plugging, and improved airway hyperresponsiveness. Mechanistically, these effects have been linked to modulation of T helper subsets—suppression of Th2/Th17 cytokine secretion, promotion of CD4^+^FoxP3^+^ Treg differentiation—and inhibition of mTOR- and NF-*κ*B–associated inflammatory signaling (Lin et al., 2020). Further evidence indicates that SGMHD mitigates airway inflammation by regulating lipid metabolism, inhibiting NOX activity, and modulating lysosomal function through downregulation of the MyD88/IKK/NF-*κ*B axis. As a consequence, systemic concentrations of IgE, C-reactive protein, IL-4, and IL-6 are diminished, accompanied by notable improvements in lung histopathology in rats sensitized with OVA (Sun et al., 2024). Within an asthma model induced by OVA combined with cold stimulation, SGMHD suppresses ASMC proliferation while promoting apoptosis, an effect associated with activation of the p70S6K/Cyclin D1 signaling axis and suppression of TAS2R10 expression. These molecular actions collectively lead to improvement in airway function–related outcomes and attenuation of key features of smooth muscle remodeling, including airway wall thickening, mucus hypersecretion, and collagen accumulation (Li et al., 2024b). Moreover, SGMHD enhances mitochondrial function by restoring the expression of mitochondrial fusion and autophagy-associated proteins (including NRF1, MIRO1, and LC3B) in exosomes derived from BALF. The activation of mitophagy contributes to reduced neutrophil infiltration and dampened airway inflammation, indirectly preventing further structural remodeling (Lu et al., 2022). Overall, current studies suggest that SGMHD may be associated with modulation of oxidative stress–related pathways such as Keap1/Nrf2/HO-1, with potential relevance to asthma-related airway mesenchymal reprogramming. Notably, the available evidence is still derived mainly from animal and cell-based experiments, and key constituent-level attribution within the formulation has not yet been robustly established.

### 6.2 BSYQF

BSYQF, a contemporary TCM formulation widely used in clinical practice, has been investigated, predominantly in OVA-driven murine asthma models, for its potential to modulate oxidative stress and improve airway inflammation and structural changes. Systems-level analyses integrating chemical profiling and transcriptomics have been used to map candidate constituents and pathway-level responses. High-performance liquid chromatography coupled with mass spectrometry (HPLC-MS) profiling has identified icariin, epimedin C, catalpol, epimedin B, and astragaloside IV as major detected constituents, often discussed as candidate contributors to the formula’s pharmacological effects (Wei et al., 2015). At the molecular systems level, transcriptomic profiling using RNA sequencing (RNA-seq) identified BSYQF-associated changes in differentially expressed genes in an OVA-triggered mouse model of asthma, with enrichment in pathways related to inflammatory signaling, oxidative-injury control, and mitochondrial function. These results suggest that BSYQF may enhance antioxidant-associated gene programs (e.g., Adipoq and HO-1) and modulate PI3K/AKT and MAPK signaling, consistent with attenuation of oxidative damage and airway remodeling–related processes (Cui et al., 2021). Functionally, BSYQF has been reported to restore the Th17/Treg equilibrium in OVA-induced asthmatic mice by dampening Th17 activity while promoting Treg function. In addition, it markedly downregulates Th2-associated cytokines such as IL-4, IL-5, and IL-13, and significantly decreases OVA-specific IgE and IgG1 concentrations, thereby alleviating persistent airway inflammation. Moreover, by suppressing profibrotic mediators (e.g., TGF-*β* and VEGF), it may curb airway mesenchymal reprogramming and thereby limit pro-remodeling structural changes. Consistent with the transcriptomic findings, integrated functional and biochemical analyses further revealed that BSYQF diminishes oxidative stress indicators in pulmonary tissue, including ROS, MDA, NO, and iNOS. In addition to elevating the GSH/GSSG ratio, the treatment promotes recovery of mitochondrial architecture in bronchial epithelial cells and boosts ATP production within lung tissue. These effects collectively reflect improved redox homeostasis, contributing to the alleviation of structural airway remodeling (Wei et al., 2015; Qin et al., 2024b; Cui et al., 2019). Compared with the bronchodilator tiotropium bromide, BSYQF demonstrated greater improvement in oxidative-stress–related readouts in these models, underscoring the value of omics-informed and systems-oriented evaluation of botanical drug formulations as complementary antioxidant therapeutic approaches for asthma treatment (Cui et al., 2019). Collectively, the evidence supports an antioxidant-linked, multi-pathway pharmacological profile for BSYQF; however, stronger conclusions will require formulation standardization and high-quality human studies.

### 6.3 Guben fangxiao decoction (GBFXD)

GBFXD is a traditional Chinese medicinal prescription formulated specifically for managing asthma. Chemical profiling of its aqueous extract has identified major bioactive metabolites including Schisandrin A, Glycyrrhizin and Astragaloside IV (Kyou-Hwan et al., 2021). These metabolites have been reported to exhibit antioxidant, anti-inflammatory, and immunoregulatory activities, but their contribution within GBFXD remains largely inferential without target-site exposure verification. Early clinical studies have suggested both efficacy and a generally acceptable safety profile of GBFXD in supporting longer-term control and reducing recurrence risk, although study scale and design vary (Xue-Jing et al., 2010). Subsequent label-free serum proteomics analyses revealed that GBFXD may promote airway epithelial repair by regulating stress responses, inflammatory processes, and epithelial cell proliferation, thereby preventing the progression of chronic airway remodeling (Xing et al., 2019). Importantly, this proteomic profiling provides system-level descriptive evidence that GBFXD modulates multiple protein networks associated with inflammation, oxidative stress responses, and tissue repair. Complementary serum metabolomics studies in asthmatic mice demonstrated that GBFXD significantly modulates key lipid metabolites involved in fatty acid metabolism and mitochondrial stress, such as acylcarnitines and phosphatidylcholines. Together with the proteomic data, these findings provide an integrative, omics-informed descriptive framework for understanding the pharmacological actions of GBFXD at the systems level. The available data implicate AMPK-associated signaling as a potential component of GBFXD’s activity, with downstream changes consistent with altered lipid metabolism, pulmonary surfactant–lipid balance, and attenuation of ongoing airway inflammation (You et al., 2021). AMPK activation is thought to confer anti-inflammatory effects by dampening mitochondrial ROS-induced endoplasmic reticulum stress, thereby preventing inflammasome activation, while also suppressing the NF-*κ*B signaling pathway (Li et al., 2015; Hattori et al., 2006). Moreover, activation of AMPK has been shown to suppress TGF-*β*1-stimulated proliferation of ASMCs, thereby playing an important part in mitigating airway remodeling (Pan et al., 2018b). Regarding its immunomodulatory potential, GBFXD has been shown to reestablish the equilibrium between T helper 17 (Th17) and Treg cells, suppress the production of pro-inflammatory mediators including IL-6, IL-1*β*, and TNF-*α*, and curb IL-13-induced mucus hypersecretion, thereby reducing inflammatory injury and mitigating airway hyperresponsiveness. In addition, it downregulates iNOS, a key enzymatic mediator linking oxidative stress with inflammatory processes (Ruan et al., 2016; Liang et al., 2020). Furthermore, GBFXD may enhance mitochondrial function and oxidative phosphorylation, improve respiratory chain activity, regulate energy metabolism, and inhibit M2 macrophage polarization. Together, these actions are consistent with improved homeostasis across the interconnected axis of mitochondrial dysfunction, oxidative stress, inflammation, and airway mesenchymal reprogramming, with reported reductions in goblet-cell proliferation, excessive mucus production, and ECM accumulation—key pathological features of airway mesenchymal reprogramming (Liu et al., 2019; Xing et al., 2021). In conclusion, the available evidence is derived mainly from multi-omics analyses and experimental models, and further work would help strengthen robustness and translational relevance.

### 6.4 Suhuang antitussive capsule

Suhuang antitussive capsule, a multi-herb botanical drug formulation used in TCM practice, has been evaluated as an adjunct intervention for cough-predominant symptoms in asthma. Clinical evidence indicates that in patients with inadequately controlled mild-to-moderate asthma, adjunctive treatment with Suhuang antitussive capsule for 14 days significantly reduced cough visual analogue scale (VAS) scores, increased cough resolution rates, and improved small airway function, accompanied by decreases in peripheral eosinophil counts, serum IgE, and major basic protein levels, without significant changes in Asthma Control Test (ACT) scores (Pan et al., 2025b). In parallel, preclinical studies using chronic OVA-induced asthma models demonstrated that Suhuang antitussive capsule reduced airway and perivascular collagen deposition, mucus hypersecretion, and airway inflammation. Mechanistically, the formulation as a whole exerts its therapeutic effects through stimulation of the aryl hydrocarbon receptor and activation of the Nrf2-dependent signaling network, which promotes the nuclear translocation of Nrf2 and subsequently enhances both the expression and enzymatic activity of pivotal antioxidant defenses, including SOD, GSH, and total antioxidant capacity (T-AOC). By orchestrating these molecular events, the intervention effectively mitigates oxidative damage and curtails the accompanying inflammatory processes (Liang et al., 2022). Moreover, by inhibiting Drp1 activation, Suhuang antitussive capsule helps preserve mitochondrial structural and functional integrity. This preservation of mitochondrial membrane potential results in a reduction of ROS production and a decrease in mtDNA release. As a result, the pro-inflammatory signaling cascades mediated by NF-*κ*B and the NLRP3 inflammasome are effectively suppressed, which in turn mitigates airway inflammatory responses in mouse models of asthma (Qin et al., 2021). In a rat model of cough variant asthma induced by OVA, administration of Suhuang antitussive capsule resulted in a marked elevation of serum *β*-hydroxybutyrate levels, subsequently activating the GSK3*β*/AMPK/Nrf2 signaling axis. This facilitates Nrf2 translocation into the nucleus, while simultaneously inhibiting NF-*κ*B signaling and curbing the activation of the NLRP3 inflammasome. These effects collectively contribute to lowering ROS generation, diminishing pro-inflammatory cytokine release, and alleviating lung tissue damage and collagen accumulation (Jiang et al., 2023). In an OVA-driven chronic asthma mouse model, treatment with a low dose of Suhuang antitussive capsule markedly alleviated airway hyperresponsiveness, curtailed inflammatory cell infiltration, and diminished mucus secretion. Additionally, it decreased TGF-*β*1 and IL-13 production, which was associated with reduced collagen accumulation and delayed progression of airway remodeling (Zhang et al., 2016). Taken together, these findings align with an airway mesenchymal reprogramming framework, but clinical confirmation using standardized preparations and predefined structural endpoints is still needed.

### 6.5 Yanghe pingchuan granules (YPG)

YPG is a botanical drug formulation developed based on years of clinical experience, specifically used to alleviate symptoms of asthma and cough (Gong et al., 2023). Analysis using ultra-performance liquid chromatography (UPLC) identified chlorogenic acid, quercetin, and naringenin as the major detected metabolites (Pan et al., 2025a). Importantly, mechanistic analyses further revealed that the botanical drug formulation as a whole induces ferroptosis in ASMCs via activation of the METTL3/p53/SLC7A11 signaling axis (Pan et al., 2025a). This ferroptosis-related process, marked by disrupted cystine transport, increased lipid peroxidation, and iron-dependent oxidative damage, promotes the clearance of hyperproliferative ASMCs and contributes to the attenuation of airway remodeling. In an OVA-induced rat asthma model, YPG was found to suppress aberrant ASMC proliferation by reducing proliferating cell nuclear antigen levels and blocking the PI3K/PKB (AKT) signaling cascade, thereby markedly ameliorating structural changes associated with airway remodeling (Pan et al., 2018a). Furthermore, YPG exhibits significant antioxidant potential by blocking the IKK/I*κ*B/NF-*κ*B signaling pathway, which results in markedly lower lipid peroxidation–derived MDA levels and diminished overproduction of NO, alongside a concomitant enhancement of SOD enzymatic activity. Such redox rebalancing leads to a marked suppression of pro-inflammatory cytokines, including IL-1*β* and IL-6, as well as a reduction in the expression of the angiogenesis-related factor VEGF and its receptor VEGFR2 (Pan et al., 2024; Gong et al., 2023). However, further studies are needed to clarify the relative contribution of ASMC ferroptosis and downstream signaling changes to YPG’s anti-remodeling effects.

### 6.6 Guben kecuan granules

Guben Kecuan Granules is an asthma treatment botanical drug formulation whose representative metabolites span various chemical categories, including flavonoids, glycosides, and coumarins. Representative metabolites identified in this formulation include liquiritigenin, codonopyrrolidium A, psoralen, glycyrrhizic acid, and schisandrol A (Luo et al., 2021). In a rat model of asthma induced by co-sensitization with OVA and alum, administration of Guben Kecuan Granules significantly lessened airway and peribronchial inflammatory cell infiltration, an effect associated with the suppression of NF-*κ*B and signal transducer and activator of transcription 3 (STAT3) pathways. Treatment with this formulation led to a marked decline in BALF concentrations of key proinflammatory cytokines, including IL-4, IL-5, IL-6, IL-10, and TNF-*α*. The treatment simultaneously restored the activities of CAT, SOD, and GSH, while lowering excessive NO and MDA accumulation, thereby alleviating oxidative stress–associated tissue damage. Furthermore, Guben Kecuan Granules downregulated the expression of MMP-9, a critical enzyme involved in pulmonary ECM turnover, and concomitantly reduced levels of its natural inhibitor, tissue inhibitor of metalloproteinase-1 (TIMP-1), thereby modulating matrix remodeling processes in lung tissue. In addition, the treatment suppressed the expression of apoptosis-promoting factors, including Bax and caspase-3, while upregulating the anti-apoptotic protein Bcl-2, thereby mitigating excessive epithelial apoptosis and contributing to the preservation of airway structural integrity. Together, these actions reduced collagen deposition and mucus-associated changes and alleviated epithelial thickening, showing a consistent improvement trend in pathological processes associated with airway mesenchymal reprogramming (Dai et al., 2025). In summary, Guben Kecuan Granules exert system-level protective effects by attenuating oxidative stress and apoptosis, thereby correcting redox imbalance and potentially helping restrain pathological processes linked to airway mesenchymal reprogramming and its associated inflammatory cascades in asthma. Nevertheless, the evidence is based mainly on an OVA/alum-driven rat model, and generalizability across asthma phenotypes remains to be confirmed.

### 6.7 Shiwei longdanhua granules (SWLDH)

SWLDH, a traditional Tibetan botanical drug formulation documented since the 19th century, has recently demonstrated efficacy in mitigating asthma-associated airway remodeling through modulation of oxidative stress via multiple synergistic mechanisms. SWLDH markedly reduces pulmonary leukocyte infiltration and bronchiolar obstruction, and it suppresses mucus hypersecretion by downregulating the key mucins MUC5AC and MUC5B. At the same time, it attenuates chronic inflammatory injury by inhibiting lipopolysaccharide (LPS)-stimulated secretion of key proinflammatory factors, including IL-6, IL-1*β*, TNF-*α*, prostaglandin E_2_, and leukotriene B_4_. SWLDH also improves airway hyper-responsiveness by lowering intracellular Ca^2+^ and acetylcholine while increasing cellular cyclic adenosine monophosphate, thus relaxing ASM. In parallel, it suppresses the profibrotic mediators TGF-*β*1 and *α*-SMA, limiting airway smooth-muscle proliferation and collagen deposition and thereby contributing to the attenuation of airway structural remodeling. These effects correlate with a restored oxidant–antioxidant balance: SWLDH elevates GSH levels and boosts the activities of GR, glutathione S-transferase (GST), CAT, SOD, thioredoxin reductase (TrxR) and thioredoxin peroxidase (TPx). By reinforcing this antioxidant defense network at the system level, SWLDH mitigates oxidative stress-induced cellular injury and significantly inhibits asthma-related airway mesenchymal reprogramming (Wei et al., 2023). However, while the findings support a beneficial effect of SWLDH on asthma-related airway mesenchymal reprogramming, further work is needed to delineate the direct versus secondary contributions of redox and inflammatory modulation (Tables 2, 3).

**TABLE 2:** Effects of traditional Chinese botanical drug formulations on oxidative stress and asthma-associated airway mesenchymal reprogramming.

Botanical drug formulation	Form	Test models		Dose	Controls used	Experimental duration	Modulation of oxidative stress markers	Associated signaling pathways	Affected processes of airway mesenchymal reprogramming	References
		*In vivo*	*In vitro*							
Shegan Mahuang Decoction	Decoction	OVA-induced Sprague-Dawley rats	​	Oral gavage administration: low dose 3.275 g/kg, medium dose 6.55 g/kg, high dose 13.1 g/kg	Normal control; OVA-induced model control; Dexamethasone (positive control)	29 days	ROS↓; NOX2 components (Ncf1/Ncf2/Ncf4/Cybb)↓	MyD88/IKK/NF-*κ*B (−)	Airway inflammation, oxidative injury (NOX-related)	Sun et al. (2024)
Bu-Shen-Yi-Qi formula	Decoction	OVA-induced BALB/c mice	​	Oral gavage administration: low dose 5 g/kg, medium dose 10 g/kg, high dose 20 g/kg	Normal control; OVA-induced model control; Tiotropium bromide (positive control)	6 weeks	ROS↓,MDA↓,NO/iNOS↓,SOD↑,GSH↑	iNOS/NF-κB (−)	Airway inflammation, smooth muscle cell proliferation, collagen deposition, airway hyperresponsiveness, goblet cell hyperplasia, mucus secretion	Cui et al. (2019)
Gu-Ben-Fang-Xiao decoction	Decoction	RSV + OVA-induced BALB/c mice	​	Oral gavage administration: low dose 12 g/kg, high dose 36 g/kg	Normal control; RSV + OVA–induced model control	83 days	iNOS↓	BAFF-mediated B-cell activation (−)	Airway inflammation; airway hyperresponsiveness	Liang et al. (2020)
Suhuang antitussive capsule	Capsule	OVA-induced Wistar rats	Bone-marrow-derived macrophage cells	*In vivo*: Oral gavage administration at 1.4 g/kg; *In vitro*: 10 mM, 20 mM, 40 mM	Normal control; OVA-induced model control; Dexamethasone (positive control)	*In vivo*: 26 days; *In vitro*: 6 h	ROS↓,Nrf2↑,HO-1↑	GSK3β/AMPK/Nrf2 axis (+); NF-κB and NLRP3 inflammasome (−)	Airway inflammation, collagen deposition	Jiang et al. (2023)
Yanghe Pingchuan granules	Granules	OVA-induced Sprague Dawley rats	​	Oral gavage administration: low dose 3.69 g/kg, medium dose 7.38 g/kg, high dose 14.76 g/kg	Normal control; OVA-induced model control; Aminophylline (positive control)	30 days	MDA↓,NO↓,SOD↑	IKK/IκB/NF-κB (−)	Airway inflammation, collagen deposition	Pan et al. (2024)
Guben Kechuan granule	Granule	OVA + AL (OH)_3_-induced rats	​	Oral gavage administration: low dose 0.405 g/kg, medium dose 0.81 g/kg, high dose 1.62 g/kg	Normal control; OVA-induced model control; Dexamethasone (positive control)	38 days	MDA↓,NO↓, SOD↑,CAT↑,GSH↑	NF-*κ*B/STAT3 (−)	Airway inflammation, mucus secretion, collagen deposition	Dai et al. (2025)
Shiwei Longdanhua Granule	Granule	LPS-induced BALB/c mice	​	Oral gavage administration: low dose 500 mg/kg, medium dose 1000 mg/kg, high dose 2000 mg/kg	Normal control; LPS-induced model control	48 h after LPS challenge	MDA↓,SOD↑,CAT↑,GSH↑,GST↑,GR↑,TrxR↑,TPx↑	Multi-pathway inhibition (−)	Airway inflammation, mucus secretion, smooth muscle cell proliferation	Wei et al. (2023)

Data were compiled from published experimental studies on traditional Chinese botanical drug formulations in asthma-related models. Symbols indicate: ↑, increase; ↓, decrease; (+), activation; (−), inhibition.

**TABLE 3:** Botanical composition (scientific names) of traditional Chinese botanical drug formulations included in this review.

Botanical drug formulation	Botanical composition (scientific names)	References
Shegan Mahuang Decoction	Belamcanda chinensis (L.) DC. Ephedra sinica Stapf Aster tataricus L.f. Tussilago farfara L. Pinellia ternata (Thunb.) Breit. Zingiber officinale Roscoe Asarum heterotropoides Fr. Schmidt var. mandshuricum (Maxim.) Kitag. Schisandra chinensis (Turcz.) Baill. Ziziphus jujuba Mill.	Sun et al. (2024)
Bu-Shen-Yi-Qi formula	Astragalus membranaceus (Fisch.) Bunge Rehmanniae Radix Libosch. Epimedium brevicornu Maxim.	Cui et al. (2019)
Gu-Ben-Fang-Xiao decoction	Astragalus membranaceus Bunge Codonopsis pilosula Nannf. Poria cocos Wolf Atractylodes macrocephala Koidz Citrus reticulata Blanco Saposhnikovia divaricata Schischk Ostrea gigas Thunberg Cryptotympana pustulata Fabricius Schisandra chinensis Baill. Magnolia denudata Desr. Glycyrrhiza uralensis Fisch.	Liang et al. (2020)
Suhuang antitussive capsule	Perilla frutescens (L.) Britt. (Folium perillae) Ephedra equisetina Bge. (Herba ephedrae) Pheretima aspergillum (E. Perrier) Cryptotympana pustulata Fabricius (Periostracum cicadae) Arctium lappa L. Schisandra chinensis (Turcz.) Baill. et Wils. Eriobotrya japonica (Thunb.) Lindl. Peucedanum praeruptorum Dunn. Perilla frutescens (L.) Britt. (Fructus perillae)	Zhang et al. (2016)
Yanghe Pingchuan granules	Ephedra sinica Stapf. Rehmannia glutinosa (Gaertn.) DC. Inula japonica Thunb. Morinda officinalis F.C.How Schisandra chinensis (Turcz.) Baill. Sinapis alba L. Draba nemorosa L. Angelica sinensis (Oliv.) Diels Platycodon grandiflorus (Jacq.) A.DC.	Pan et al. (2024)
Guben Kechuan granule	Codonopsis pilosula (Franch.) Nannf. Atractylodes macrocephala Koidz. Schisandra chinensis (Turcz.) Baill. Ophiopogon japonicus (L. f.) Ker Gawl. Glycyrrhiza uralensis Fisch. Psoralea corylifolia Linn. Poria cocos (Schw.) Wolf	Dai et al. (2025)
Shiwei Longdanhua Granule	Gentiana scabra Bunge Rhododendron anthopogonoides Maxim. Fritillaria cirrhosa D. Don Corydalis hendersonii Hemsl Berberis kansuensis Schneid. Phlomis younghusbandii Mukerjee Codonopsis convolvulacea Kurz Przewalskia tangutica Maxim. Glycyrrhiza uralensis Fisch.	Wei et al. (2023)

Botanical compositions are presented using accepted scientific names as reported in the cited references.

## 7 Conclusion and perspectives

Asthma-related airway mesenchymal reprogramming refers to persistent pathological alterations in the phenotype, function, and intercellular interactions of airway mesenchymal cells under asthmatic conditions, which drive airway structural remodeling and exacerbate chronic inflammation (Mottais et al., 2023; Brasier and Boldogh, 2019). Oxidative stress plays a pivotal role in this process by activating inflammatory signaling pathways, promoting EMT, aggravating ASM dysfunction, and impairing endogenous antioxidant defense systems. This review systematically summarizes the potential advantages of TCM in modulating asthma-related airway mesenchymal reprogramming and oxidative stress; however, substantial challenges remain in translating these findings from basic research into clinical application.

Notably, asthma is a highly heterogeneous chronic inflammatory airway disease that can be classified into eosinophilic, neutrophilic, mixed granulocytic, and paucigranulocytic phenotypes based on sputum inflammatory cell profiles (Plavsic et al., 2024), among which eosinophilic and neutrophilic asthma are the most prevalent. Eosinophilic asthma is characterized by eosinophilic infiltration and a predominant type 2 immune response, whereas neutrophilic asthma is driven by neutrophils, is closely associated with activation of Th1/Th17 signaling pathways, and often exhibits poor responsiveness to glucocorticoid therapy (Carr et al., 2018; Ray and Kolls, 2017). Clinical evidence further indicates that different asthma phenotypes differ substantially in airway remodeling features, immunological mechanisms, and therapeutic responses (Kuruvilla et al., 2019). However, most studies included in this review are based on OVA-induced eosinophilic asthma models, while evidence related to neutrophilic asthma remains limited. This model bias restricts, to some extent, the extrapolation of current findings to different asthma phenotypes and their clinical applicability.

In addition, most existing clinical studies have evaluated TCM as botanical drug formulations administered as adjuncts to standard therapy. For example, a multicenter, randomized, double-blind, placebo-controlled trial demonstrated that the addition of Suhuang antitussive capsule to inhaled corticosteroids combined with long-acting β_2_-agonists significantly improved cough-related scores and reduced peripheral blood eosinophil counts and IgE levels, with a favorable safety profile (Pan et al., 2025b). Furthermore, a systematic review and meta-analysis including 992 pediatric asthma patients reported that botanical drug formulations combined with conventional therapy improved overall clinical efficacy and reduced serum total IgE levels, recurrence rates, and the incidence of adverse events (Kyou-Hwan et al., 2021). Overall, current clinical evidence suggests that botanical drug formulations have potential therapeutic value in asthma management; however, the limited number of randomized controlled trials and the generally low level of evidence mean that the causal relationships between efficacy and safety remain insufficiently established, thereby limiting broader acceptance in clinical guidelines and standardized application.

Taken together, multiple challenges persist in translating findings from basic research into clinical practice. In addition to asthma heterogeneity and insufficient high-quality clinical evidence, unclear pharmacokinetic characteristics of bioactive metabolites and inadequate standardization of botanical drug formulations further hinder translational progress. Therefore, future studies may integrate spatial omics and machine learning approaches to combine clinical phenotypic indicators with multi-omics features, enabling refined asthma endotyping and more precise characterization of disease heterogeneity (Sakkal et al., 2026; Ray et al., 2022). Meanwhile, the application of artificial intelligence to establish large-scale, multicenter clinical databases may improve data quality and representativeness, thereby facilitating the development of precision medicine. Moreover, the establishment of pharmacokinetic-pharmacodynamic integrated analysis frameworks guided by key bioactive metabolites, together with strengthened quality control systems spanning from raw materials to finished products, may promote the standardized and modernized application of botanical drug formulations in asthma treatment (Wang et al., 2026).

Overall, oxidative stress and its associated airway mesenchymal reprogramming play critical roles in the initiation and progression of asthma. Through multi-target and multi-level regulatory mechanisms, botanical drug formulations offer a novel perspective for intervening in this complex pathological process. Although current evidence remains subject to certain limitations, continued advances in research methodologies and technological approaches are expected to further deepen mechanistic understanding and promote sustained progress in this field.

## Glossary

ACT: Asthma Control Test
AMPK: AMP-activated protein kinase
ASM: Airway smooth muscle
ASMCs: airway smooth muscle cells
ATP: adenosine triphosphate
BALF: bronchoalveolar lavage fluid
BSYQF: Bushen Yiqi Formula
CAT: catalase
CO: carbon monoxide
COX-2: cyclooxygenase-2
Drp1: dynamin-related protein 1
ECM: extracellular matrix
EMT: epithelial-mesenchymal transition
EPO: eosinophil peroxidase
ERK1/2: extracellular signal-regulated kinases 1 and 2
GBFXD: Guben Fangxiao Decoction
GPx: glutathione peroxidase
GR: glutathione reductase
GSH: glutathione
GST: glutathione S-transferase
HO-1: heme oxygenase-1
HPLC-MS: high-performance liquid chromatography coupled with mass spectrometry
HPLC-UV: High-Performance Liquid Chromatography with Ultraviolet Detection
IgE: immunoglobulin E
IKK: I*κ*B kinase
IL-13: interleukin-13
IL-1*β*: Interleukin-1 beta
IL-6: interleukin-6
IL-8: interleukin-8
iNOS: inducible nitric oxide synthase
I*κ*B*α*: inhibitor of kappa B alpha
JNK: c-Jun N-terminal kinase
Keap1: Kelch-like ECH-associated protein 1
LPS: lipopolysaccharide
MAPK: mitogen-activated protein kinase
MDA: Malondialdehyde
MMP-9: matrix metalloproteinase-9
MPO: myeloperoxidase
mtDNA: mitochondrial DNA
NAC: N-acetylcysteine
NADPH: reduced nicotinamide adenine dinucleotide phosphate
NF-*κ*B: nuclear factor kappa B
NLRP3: NOD-like receptor family pyrin domain containing 3
NOX: NADPH oxidase
NOX4: NADPH oxidase 4
NQO1: NAD(P)H quinone dehydrogenase 1
Nrf2: nuclear factor erythroid 2-related factor 2
OVA: ovalbumin
PI3K/Akt: Phosphoinositide 3-Kinase/Protein Kinase B
RNA-seq: RNA sequencing
RNS: reactive nitrogen species
ROS: reactive oxygen species
SGMHD: Shegan Mahuang Decoction
SOD: superoxide dismutase
STAT3: signal transducer and activator of transcription 3
SWLDH: Shiwei Longdanhua Granules
T-AOC: total antioxidant capacity
TCM: traditional Chinese medicine
TGF-*β*: transforming growth factor beta
TGF-*β*1: transforming growth factor beta 1
Th17: T helper 17
Th2: T helper 2
TIMP-1: tissue inhibitor of metalloproteinase-1
TNF-*α*: tumor necrosis factor-alpha
TPx: thioredoxin peroxidase
Treg: regulatory T cell
TrxR: thioredoxin reductase
UPLC: Ultra-performance liquid chromatography
VAS: visual analogue scale
VEGF: vascular endothelial growth factor
YPG: Yanghe Pingchuan Granules
ZO-1: zonula occludens-1
*α*-SMA: alpha-smooth muscle actin
15-LOX: 15-lipoxygenase
**8-iso-PGF**_**2**_ ***α***: 8-iso-prostaglandin F_2_ *α*
8-OHdG: 8-hydroxy-2′-deoxyguanosine

## References

[B1] Albano G. D. Gagliardo R. P. Montalbano A. M. Profita M. (2022). Overview of the mechanisms of oxidative stress: impact in inflammation of the airway diseases. Antioxidants (Basel) 11, 2237. 10.3390/antiox11112237 36421423 PMC9687037

[B2] Asatiani N. Sapojnikova N. Kartvelishvili T. Asanishvili L. Sichinava N. Chikovani Z. (2025). Blood catalase, superoxide dismutase, and glutathione peroxidase activities in Alcohol- and opioid-addicted patients. Med. Kaunas. 61, 204. 10.3390/medicina61020204 40005321 PMC11857377

[B3] Audousset C. Mcgovern T. Martin J. G. (2021). Role of Nrf2 in disease: novel molecular mechanisms and therapeutic approaches - pulmonary disease/Asthma. Front. Physiol. 12, 727806. 10.3389/fphys.2021.727806 34658913 PMC8511424

[B4] Barnes P. J. Karin M. (1997). Nuclear factor-kappaB: a pivotal transcription factor in chronic inflammatory diseases. N. Engl. J. Med. 336, 1066–1071. 10.1056/NEJM199704103361506 9091804

[B5] Bass-Stringer S. Bernardo B. C. May C. N. Thomas C. J. Weeks K. L. Mcmullen J. R. (2018). Adeno-Associated virus gene therapy: translational progress and future prospects in the treatment of heart failure. Heart Lung Circ. 27, 1285–1300. 10.1016/j.hlc.2018.03.005 29703647

[B6] Bondonno N. P. Parmenter B. H. Thompson A. S. Jennings A. Murray K. Rasmussen D. B. (2024). Flavonoid intakes, chronic obstructive pulmonary disease, adult asthma, and lung function: a cohort study in the UK Biobank. Am. J. Clin. Nutr. 120, 1195–1206. 10.1016/j.ajcnut.2024.08.032 39222688 PMC11600086

[B7] Boutten A. Goven D. Artaud-Macari E. Bonay M. (2011). Protective role of Nrf2 in the lungs against oxidative airway diseases. Med. Sci. Paris. 27, 966–972. 10.1051/medsci/20112711012 22130023

[B8] Braicu C. Buse M. Busuioc C. Drula R. Gulei D. Raduly L. (2019). A comprehensive review on MAPK: a promising therapeutic target in cancer. Cancers (Basel) 11 (10), 1618. 10.3390/cancers11101618 31652660 PMC6827047

[B9] Brasier A. R. Boldogh I. (2019). Targeting inducible epigenetic reprogramming pathways in chronic airway remodeling. Drugs Context 8. 10.7573/dic.2019-8-3 31692901 PMC6821469

[B10] Burton G. J. Jauniaux E. (2011). Oxidative stress. Best. Pract. Res. Clin. Obstet. Gynaecol. 25, 287–299. 10.1016/j.bpobgyn.2010.10.016 21130690 PMC3101336

[B11] Camps M. Rückle T. Ji H. Ardissone V. Rintelen F. Shaw J. (2005). Blockade of PI3Kgamma suppresses joint inflammation and damage in mouse models of rheumatoid arthritis. Nat. Med. 11, 936–943. 10.1038/nm1284 16127437

[B12] Cano A. Pérez-Moreno M. A. Rodrigo I. Locascio A. Blanco M. J. Del Barrio M. G. (2000). The transcription factor snail controls epithelial-mesenchymal transitions by repressing E-cadherin expression. Nat. Cell Biol. 2, 76–83. 10.1038/35000025 10655586

[B13] Carr T. F. Zeki A. A. Kraft M. (2018). Eosinophilic and noneosinophilic asthma. Am. J. Respir. Crit. Care Med. 197, 22–37. 10.1164/rccm.201611-2232PP 28910134 PMC5765385

[B14] Chen M. Shi J. T. Lv Z. Q. Huang L. J. Lin X. L. Zhang W. (2014). Triptolide inhibits TGF-β1 induced proliferation and migration of rat airway smooth muscle cells by suppressing NF-κB but not ERK1/2. Immunology 144, 486–494. 10.1111/imm.12396 25267491 PMC4557685

[B15] Chen M. Lv Z. Zhang W. Huang L. Lin X. Shi J. (2015). Triptolide suppresses airway goblet cell hyperplasia and Muc5ac expression *via* NF-κB in a murine model of asthma. Mol. Immunol. 64, 99–105. 10.1016/j.molimm.2014.11.001 25466609

[B16] Chen Y. Wang J. Zhang Y. Zhang Z. Chen H. Hu J. (2025). Investigation of key ferroptosis-associated genes and potential therapeutic drugs for asthma based on machine learning and regression models. Sci. Rep. 15, 20342. 10.1038/s41598-025-06122-6 40715138 PMC12297556

[B17] Cui J. Xu F. Tang Z. Wang W. Hu L. L. Yan C. (2019). Bu-Shen-Yi-Qi formula ameliorates airway remodeling in murine chronic asthma by modulating airway inflammation and oxidative stress in the lung. Biomed. Pharmacother. 112, 108694. 10.1016/j.biopha.2019.108694 30798140

[B18] Cui J. Lv Z. Teng F. Yi L. Tang W. Wang W. (2021). RNA-seq expression analysis of chronic asthmatic mice with bu-shen-yi-qi formula treatment and prediction of regulated gene targets of anti-airway remodeling. Evid. Based Complement. Altern. Med. 2021, 3524571. 10.1155/2021/3524571 33531915 PMC7834776

[B19] Dai R. Niu M. Wang N. Wang Y. (2021). Syringin alleviates ovalbumin-induced lung inflammation in BALB/c mice asthma model *via* NF-κB signaling pathway. Environ. Toxicol. 36, 433–444. 10.1002/tox.23049 33146439

[B20] Dai C. Liu D. Qin C. Fang J. Cheng G. Xu C. (2025). Guben kechuan granule attenuates bronchial asthma by inhibiting NF-κB/STAT3 signaling pathway-mediated apoptosis. J. Ethnopharmacol. 340, 119124. 10.1016/j.jep.2024.119124 39694430

[B21] Danke N. A. Kwok W. W. (2003). HLA class II-restricted CD4+ T cell responses directed against influenza viral antigens postinfluenza vaccination. J. Immunol. 171, 3163–3169. 10.4049/jimmunol.171.6.3163 12960344

[B22] Diaz J. V. Anthon G. E. Barrett D. M. (2009). Conformational changes in serum pectins during industrial tomato paste production. J. Agric. Food Chem. 57, 8453–8458. 10.1021/jf901207w 19702334

[B23] Doeing D. C. Solway J. (2013). Airway smooth muscle in the pathophysiology and treatment of asthma. J. Appl. Physiol. (1985) 114, 834–843. 10.1152/japplphysiol.00950.2012 23305987 PMC3633438

[B24] Dougherty S. M. Mazhawidza W. Bohn A. R. Robinson K. A. Mattingly K. A. Blankenship K. A. (2006). Gender difference in the activity but not expression of estrogen receptors alpha and beta in human lung adenocarcinoma cells. Endocr. Relat. Cancer 13, 113–134. 10.1677/erc.1.01118 16601283 PMC1472635

[B25] Duchesne M. Okoye I. Lacy P. (2022). Epithelial cell alarmin cytokines: frontline mediators of the asthma inflammatory response. Front. Immunol. 13, 975914. 10.3389/fimmu.2022.975914 36311787 PMC9616080

[B26] Ge D. Chen Q. Xie X. Li Q. Yang Y. (2024). Unveiling the potent effect of vitamin D: harnessing Nrf2/HO-1 signaling pathways as molecular targets to alleviate urban particulate matter-induced asthma inflammation. BMC Pulm. Med. 24, 55. 10.1186/s12890-024-02869-2 38273268 PMC10809564

[B27] Geng Q. Yan L. Shi C. Zhang L. Li L. Lu P. (2024). Therapeutic effects of flavonoids on pulmonary fibrosis: a preclinical meta-analysis. Phytomedicine 132, 155807. 10.1016/j.phymed.2024.155807 38876010

[B28] Ghafouri-Fard S. Abak A. Tondro Anamag F. Shoorei H. Majidpoor J. Taheri M. (2021). The emerging role of non-coding RNAs in the regulation of PI3K/AKT pathway in the carcinogenesis process. Biomed. Pharmacother. 137, 111279. 10.1016/j.biopha.2021.111279 33493969

[B29] Gong C. Pan L. Jiang Y. Sun Y. Han Y. Wang D. (2023). Investigating the mechanism of action of yanghe pingchuan granule in the treatment of bronchial asthma based on bioinformatics and experimental validation. Heliyon 9, e21936. 10.1016/j.heliyon.2023.e21936 38027735 PMC10654227

[B30] Hackett T. L. (2012). Epithelial-mesenchymal transition in the pathophysiology of airway remodelling in asthma. Curr. Opin. Allergy Clin. Immunol. 12, 53–59. 10.1097/ACI.0b013e32834ec6eb 22217512

[B31] Hackett T. L. Warner S. M. Stefanowicz D. Shaheen F. Pechkovsky D. V. Murray L. A. (2009). Induction of epithelial-mesenchymal transition in primary airway epithelial cells from patients with asthma by transforming growth factor-beta1. Am. J. Respir. Crit. Care Med. 180, 122–133. 10.1164/rccm.200811-1730OC 19406982

[B157] Han X. Hu S. Yang Q. Sang X. Tang D. Cao G. (2022). Paeoniflorin ameliorates airway inflammation and immune response in ovalbumin induced asthmatic mice: From oxidative stress to autophagy. Phytomedicine 96, 153835. 10.1016/j.phymed.2021.153835 34799185

[B160] Hanna D. A. Khalaf M. M. Abo-Saif A. A. (2019). Polydatin protects against ovalbumin-induced bronchial asthma in rats; involvement of urocortin and surfactant-D expression. Immunopharmacol. Immunotoxicol. 41 (3), 403–412. 10.1080/08923973.2018.1536985 30422021

[B32] Hattori Y. Suzuki K. Hattori S. Kasai K. (2006). Metformin inhibits cytokine-induced nuclear factor kappaB activation *via* AMP-activated protein kinase activation in vascular endothelial cells. Hypertension 47, 1183–1188. 10.1161/01.HYP.0000221429.94591.72 16636195

[B33] He S. Chen M. Lin X. Lv Z. Liang R. Huang L. (2020). Triptolide inhibits PDGF-induced proliferation of ASMCs through G0/G1 cell cycle arrest and suppression of the AKT/NF-κB/cyclinD1 signaling pathway. Eur. J. Pharmacol. 867, 172811. 10.1016/j.ejphar.2019.172811 31756335

[B34] Hennessy B. T. Smith D. L. Ram P. T. Lu Y. Mills G. B. (2005). Exploiting the PI3K/AKT pathway for cancer drug discovery. Nat. Rev. Drug Discov. 4, 988–1004. 10.1038/nrd1902 16341064

[B35] Hirota N. Martin J. G. (2013). Mechanisms of airway remodeling. Chest 144, 1026–1032. 10.1378/chest.12-3073 24008953

[B36] Ho W. E. Cheng C. Peh H. Y. Xu F. Tannenbaum S. R. Ong C. N. (2012). Anti-malarial drug artesunate ameliorates oxidative lung damage in experimental allergic asthma. Free Radic. Biol. Med. 53, 498–507. 10.1016/j.freeradbiomed.2012.05.021 22634146

[B37] Hough K. P. Curtiss M. L. Blain T. J. Liu R. M. Trevor J. Deshane J. S. (2020). Airway remodeling in asthma. Front. Med. (Lausanne) 7, 191. 10.3389/fmed.2020.00191 32509793 PMC7253669

[B38] Hsieh H. L. Liu S. H. Chen Y. L. Huang C. Y. Wu S. J. (2022). Astragaloside IV suppresses inflammatory response *via* suppression of NF-κB, and MAPK signalling in human bronchial epithelial cells. Arch. Physiol. Biochem. 128, 757–766. 10.1080/13813455.2020.1727525 32057253

[B39] Hu X. Shen Y. Zhao Y. Wang J. Zhang X. Tu W. (2021). Epithelial aryl hydrocarbon receptor protects from mucus production by inhibiting ROS-triggered NLRP3 inflammasome in asthma. Front. Immunol. 12, 767508. 10.3389/fimmu.2021.767508 34868022 PMC8634667

[B40] Hu L. Li L. Yan C. Cao Y. Duan X. Sun J. (2023). Baicalin inhibits airway smooth muscle cells proliferation through the RAS signaling pathway in murine asthmatic airway remodeling model. Oxid. Med. Cell Longev. 2023, 4144138. 10.1155/2023/4144138 36814956 PMC9940961

[B41] Huang Y. Qiu C. (2022). Research advances in airway remodeling in asthma: a narrative review. Ann. Transl. Med. 10, 1023. 10.21037/atm-22-2835 36267708 PMC9577744

[B42] Huang Y. Jiang W. Zhou R. (2024). DAMP sensing and Sterile inflammation: intracellular, intercellular and inter-organ pathways. Nat. Rev. Immunol. 24, 703–719. 10.1038/s41577-024-01027-3 38684933

[B43] Ilari S. Proietti S. Milani F. Vitiello L. Muscoli C. Russo P. (2025). Dietary patterns, oxidative stress, and early inflammation: a systematic review and meta-analysis comparing mediterranean, vegan, and vegetarian diets. Nutrients 17, 548. 10.3390/nu17030548 39940408 PMC11819869

[B44] Jiang J. Wang K. Chen Y. Chen H. Nice E. C. Huang C. (2017). Redox regulation in tumor cell epithelial-mesenchymal transition: molecular basis and therapeutic strategy. Signal Transduct. Target Ther. 2, 17036. 10.1038/sigtrans.2017.36 29263924 PMC5661624

[B45] Jiang H. Bai Z. Ou Y. Liu H. Si Z. Liu Y. (2023). β-Hydroxybutyric acid upregulated by suhuang antitussive capsule ameliorates cough variant asthma through GSK3β/AMPK-Nrf2 signal axis. J. Ethnopharmacol. 307, 116013. 10.1016/j.jep.2022.116013 36586526

[B46] Jiang Y. Nguyen T. V. Jin J. Yu Z. N. Song C. H. Chai O. H. (2024). Tectorigenin inhibits oxidative stress by activating the Keap1/Nrf2/HO-1 signaling pathway in Th2-mediated allergic asthmatic mice. Free Radic. Biol. Med. 212, 207–219. 10.1016/j.freeradbiomed.2023.12.031 38147892

[B47] Juan C. A. Pérez de La Lastra J. M. Plou F. J. Pérez-Lebeña E. (2021). The chemistry of reactive oxygen species (ROS) revisited: outlining their role in biological macromolecules (DNA, lipids and proteins) and induced pathologies. Int. J. Mol. Sci. 22, 4642. 10.3390/ijms22094642 33924958 PMC8125527

[B48] Juergens U. R. (2014). Anti-inflammatory properties of the monoterpene 1.8-cineole: current evidence for co-medication in inflammatory airway diseases. Drug Res. (Stuttg) 64, 638–646. 10.1055/s-0034-1372609 24831245

[B49] Khan N. Syed D. N. Ahmad N. Mukhtar H. (2013). Fisetin: a dietary antioxidant for health promotion. Antioxid. Redox Signal 19, 151–162. 10.1089/ars.2012.4901 23121441 PMC3689181

[B50] Kim S. R. Kim D. I. Kim S. H. Lee H. Lee K. S. Cho S. H. (2014). NLRP3 inflammasome activation by mitochondrial ROS in bronchial epithelial cells is required for allergic inflammation. Cell Death Dis. 5, e1498. 10.1038/cddis.2014.460 25356867 PMC4237270

[B51] Kizhner V. Xu D. Krespi Y. P. (2011). A new tool measuring oral malodor quality of life. Eur. Arch. Otorhinolaryngol. 268, 1227–1232. 10.1007/s00405-011-1518-x 21327730

[B52] Koundouros N. Poulogiannis G. (2018). Phosphoinositide 3-Kinase/Akt signaling and redox metabolism in cancer. Front. Oncol. 8, 160. 10.3389/fonc.2018.00160 29868481 PMC5968394

[B53] Krymskaya V. P. Ammit A. J. Hoffman R. K. Eszterhas A. J. Panettieri R. A. JR (2001). Activation of class IA PI3K stimulates DNA synthesis in human airway smooth muscle cells. Am. J. Physiol. Lung Cell Mol. Physiol. 280, L1009–L1018. 10.1152/ajplung.2001.280.5.L1009 11290526

[B54] Kuruvilla M. E. Lee F. E. Lee G. B. (2019). Understanding asthma phenotypes, endotypes, and mechanisms of disease. Clin. Rev. Allergy Immunol. 56, 219–233. 10.1007/s12016-018-8712-1 30206782 PMC6411459

[B55] Kyou-Hwan H. Ki Haeng C. Cui S. Q. Lily L. Jaejong K. (2021). Effectiveness and safety of traditional Chinese herbs in children with cough variant asthma: a systematic review and Meta-analysi. J. Tradit. Chin. Med. 41, 661–668. 10.19852/j.cnki.jtcm.2021.05.001 34708623

[B56] Lee I. T. Yang C. M. (2012). Role of NADPH oxidase/ROS in pro-inflammatory mediators-induced airway and pulmonary diseases. Biochem. Pharmacol. 84, 581–590. 10.1016/j.bcp.2012.05.005 22587816

[B57] Lee D. C. Oh J. M. Choi H. Kim S. W. Kim S. W. Kim B. G. (2021). Eupatilin inhibits reactive oxygen species generation *via* Akt/NF-κB/MAPK signaling pathways in particulate matter-exposed human bronchial epithelial cells. Toxics 9, 38. 10.3390/toxics9020038 33670750 PMC7922545

[B58] Lei Z. L. Liu X. J. Ma J. X. Zhu J. (2009). Effects of matrine on airway inflammation and early airway remodeling in asthmatic mice. Zhonghua Jie He He Hu Xi Za Zhi 32, 165–170. 19575933

[B59] Li J. Wang Y. Wang Y. Wen X. Ma X. N. Chen W. (2015). Pharmacological activation of AMPK prevents Drp1-mediated mitochondrial fission and alleviates endoplasmic reticulum stress-associated endothelial dysfunction. J. Mol. Cell Cardiol. 86, 62–74. 10.1016/j.yjmcc.2015.07.010 26196303

[B60] Li J. Liu J. Yue W. Xu K. Cai W. Cui F. (2020). Andrographolide attenuates epithelial-mesenchymal transition induced by TGF-β1 in alveolar epithelial cells. J. Cell Mol. Med. 24, 10501–10511. 10.1111/jcmm.15665 32705806 PMC7521220

[B61] Li L. Zhang W. Qiu C. (2022). Cellular sources of airway smooth muscle cells in asthmatic airway remodeling and their clinical relevance: a narrative review. Ann. Transl. Med. 10, 838. 10.21037/atm-22-3219 36034982 PMC9403924

[B62] Li M. Jia D. Li J. Li Y. Wang Y. Wang Y. (2024a). Scutellarin alleviates ovalbumin-induced airway remodeling in mice and TGF-β-Induced pro-fibrotic phenotype in human bronchial epithelial cells *via* MAPK and Smad2/3 signaling pathways. Inflammation 47, 853–873. 10.1007/s10753-023-01947-7 38168709 PMC11147947

[B63] Li Q. Shan X. Yuan Y. Ye W. Fang X. (2024b). Shegan-mahuang decoction ameliorates cold-induced asthma *via* regulating the proliferation and apoptosis of airway smooth muscle cells through TAS2R10: an *in vivo* and *in vitro* study. J. Ethnopharmacol. 334, 118504. 10.1016/j.jep.2024.118504 38950796

[B64] Liang Z. Q. Tu P. C. Ji J. J. Xing Q. Q. Zhao X. (2020). Gu-ben-fang-xiao attenuates allergic airway inflammation by inhibiting BAFF-mediated B cell activation. Biomed. Pharmacother. 132, 110801. 10.1016/j.biopha.2020.110801 33049582

[B65] Liang R. Tong X. Dong Z. Qin W. Fan L. Bai Z. (2022). Suhuang antitussive capsule ameliorates post-infectious cough in mice through AhR-Nrf2 pathway. J. Ethnopharmacol. 283, 114664. 10.1016/j.jep.2021.114664 34555451

[B66] Liao W. Foo H. Y. C. Tran T. N. Q. Chai C. L. L. Wong W. S. F. (2023). Calcaratarin D, a labdane diterpenoid, attenuates mouse asthma *via* modulating alveolar macrophage function. Br. J. Pharmacol. 180, 1056–1071. 10.1111/bph.15993 36440573

[B67] Lin C. C. Wang Y. Y. Chen S. M. Liu Y. T. Li J. Q. Li F. (2020). Shegan-mahuang decoction ameliorates asthmatic airway hyperresponsiveness by downregulating Th2/Th17 cells but upregulating CD4+FoxP3+ tregs. J. Ethnopharmacol. 253, 112656. 10.1016/j.jep.2020.112656 32035217

[B68] Liu Y. N. Zha W. J. Ma Y. Chen F. F. Zhu W. Ge A. (2015). Galangin attenuates airway remodelling by inhibiting TGF-β1-mediated ROS generation and MAPK/akt phosphorylation in asthma. Sci. Rep. 5, 11758. 10.1038/srep11758 26156213 PMC4496730

[B69] Liu L. W. Xing Q. Q. Zhao X. Tan M. Lu Y. Dong Y. M. (2019). Proteomic analysis provides insights into the therapeutic effect of GU-BEN-FANG-XIAO decoction on a persistent asthmatic mouse model. Front. Pharmacol. 10, 441. 10.3389/fphar.2019.00441 31133848 PMC6514195

[B70] Liu Y. Yin Q. Liu B. Lu Z. Liu M. Meng L. (2024). Fisetin reduces ovalbumin-triggered airway remodeling by preventing phenotypic switching of airway smooth muscle cells. Respir. Res. 25, 370. 10.1186/s12931-024-03005-8 39402516 PMC11479573

[B71] Loboda A. Damulewicz M. Pyza E. Jozkowicz A. Dulak J. (2016). Role of Nrf2/HO-1 system in development, oxidative stress response and diseases: an evolutionarily conserved mechanism. Cell Mol. Life Sci. 73, 3221–3247. 10.1007/s00018-016-2223-0 27100828 PMC4967105

[B72] Lu H. N. Fu Z. Chen X. Yang M. M. Chen Y. F. Yang L. L. (2022). Shegan mahuang decoction may reduce airway inflammation in neutrophilic asthmatic mice by improving the mitochondrial function of bronchoalveolar lavage fluid exosomes. Evid. Based Complement. Altern. Med. 2022, 2477510. 10.1155/2022/2477510 36578267 PMC9792254

[B73] Luo Z. Yu G. Wang W. Sun R. Zhang B. Wang J. (2021). Integrated systems pharmacology and surface plasmon resonance approaches to reveal the synergistic effect of multiple components of gu-ben-ke-chuan decoction on chronic bronchitis. J. Inflamm. Res. 14, 1455–1471. 10.2147/JIR.S303530 33883922 PMC8055291

[B74] Mabalirajan U. Dinda A. K. Sharma S. K. Ghosh B. (2009). Esculetin restores mitochondrial dysfunction and reduces allergic asthma features in experimental murine model. J. Immunol. 183, 2059–2067. 10.4049/jimmunol.0900342 19570833

[B75] Mabalirajan U. Ahmad T. Rehman R. Leishangthem G. D. Dinda A. K. Agrawal A. (2013). Baicalein reduces airway injury in allergen and IL-13 induced airway inflammation. PLoS One 8, e62916. 10.1371/journal.pone.0062916 23646158 PMC3639905

[B76] Manning B. D. Toker A. (2017). AKT/PKB signaling: navigating the network. Cell 169, 381–405. 10.1016/j.cell.2017.04.001 28431241 PMC5546324

[B155] Meng Z. Chen H. Deng C. Meng S. (2022). Potential cellular endocrinology mechanisms underlying the effects of Chinese herbal medicine therapy on asthma. Front Endocrinol. (Lausanne) 13, 916328. 10.3389/fendo.2022.916328 36051395 PMC9424672

[B77] Michaeloudes C. Abubakar-Waziri H. Lakhdar R. Raby K. Dixey P. Adcock I. M. (2022). Molecular mechanisms of oxidative stress in asthma. Mol. Asp. Med. 85, 101026. 10.1016/j.mam.2021.101026 34625291

[B78] Mims J. W. (2015). Asthma: definitions and pathophysiology. Int. Forum Allergy Rhinol. 5 (Suppl. 1), S2–S6. 10.1002/alr.21609 26335832

[B79] Ming X. Yu X. Li J. Wang J. Zheng J. Xiong L. (2022). Salidroside attenuates airway inflammation and remodeling *via* the miR-323-3p/SOCS5 axis in asthmatic mice. Int. Arch. Allergy Immunol. 183, 424–434. 10.1159/000520444 34856542

[B80] Mishra V. Banga J. Silveyra P. (2018). Oxidative stress and cellular pathways of asthma and inflammation: therapeutic strategies and pharmacological targets. Pharmacol. Ther. 181, 169–182. 10.1016/j.pharmthera.2017.08.011 28842273 PMC5743757

[B81] Mittal M. Siddiqui M. R. Tran K. Reddy S. P. Malik A. B. (2014). Reactive oxygen species in inflammation and tissue injury. Antioxid. Redox Signal 20, 1126–1167. 10.1089/ars.2012.5149 23991888 PMC3929010

[B82] Mokra D. Mokry J. Barosova R. Hanusrichterova J. (2023). Advances in the use of N-Acetylcysteine in chronic respiratory diseases. Antioxidants (Basel) 12, 1713. 10.3390/antiox12091713 37760016 PMC10526097

[B83] Mottais A. Riberi L. Falco A. Soccal S. Gohy S. de Rose V. (2023). Epithelial-mesenchymal transition mechanisms in chronic airway diseases: a common process to target? Int. J. Mol. Sci. 24, 12412. 10.3390/ijms241512412 37569787 PMC10418908

[B84] Nakanishi A. Wada Y. Kitagishi Y. Matsuda S. (2014). Link between PI3K/AKT/PTEN pathway and NOX proteinin diseases. Aging Dis. 5, 203–211. 10.14336/AD.2014.0500203 24900943 PMC4037312

[B85] Nikitovic D. Corsini E. Kouretas D. Tsatsakis A. Tzanakakis G. (2013). ROS-major mediators of extracellular matrix remodeling during tumor progression. Food Chem. Toxicol. 61, 178–186. 10.1016/j.fct.2013.06.013 23792086

[B86] O'Rourke T. W. Doudican N. A. Mackereth M. D. Doetsch P. W. Shadel G. S. (2002). Mitochondrial dysfunction due to oxidative mitochondrial DNA damage is reduced through cooperative actions of diverse proteins. Mol. Cell Biol. 22, 4086–4093. 10.1128/mcb.22.12.4086-4093.2002 12024022 PMC133882

[B87] O'Sullivan M. J. Jang J. H. Panariti A. Bedrat A. Ijpma G. Lemos B. (2021). Airway epithelial cells drive airway smooth muscle cell phenotype switching to the proliferative and pro-inflammatory phenotype. Front. Physiol. 12, 687654. 10.3389/fphys.2021.687654 34295265 PMC8290262

[B88] Pan L. Y. Han Y. Q. Wang Y. Z. Chen Q. Q. Wu Y. Sun Y. (2018a). Mechanism of yanghe pingchuan granules treatment for airway remodeling in asthma. Drug Des. Devel Ther. 12, 1941–1951. 10.2147/DDDT.S159428 29983548 PMC6027695

[B89] Pan Y. Liu L. Li S. Wang K. Ke R. Shi W. (2018b). Activation of AMPK inhibits TGF-β1-induced airway smooth muscle cells proliferation and its potential mechanisms. Sci. Rep. 8, 3624. 10.1038/s41598-018-21812-0 29483552 PMC5827654

[B90] Pan S. Conaway S. JR. Deshpande D. A. (2019). Mitochondrial regulation of airway smooth muscle functions in health and pulmonary diseases. Arch. Biochem. Biophys. 663, 109–119. 10.1016/j.abb.2019.01.002 30629957 PMC6377851

[B91] Pan L. Gong C. Chen Y. Jiang Y. Sun Y. He B. (2024). Yanghe pingchuan granules mitigates oxidative stress and inflammation in a bronchial asthma rat model: role of the IKK/IκB/NF-κB signalling pathway. Ann. Med. Surg. (Lond) 86, 212–218. 10.1097/MS9.0000000000001553 38222706 PMC10783385

[B92] Pan L. He B. Han Y. Yuan D. Duan X. Wang Y. (2025a). Yanghe pingchuan granules induce ferroptosis in airway smooth muscle cells to improve bronchial asthma *via* the METTL3/P53/SLC7A11 signaling pathway. Phytomedicine 139, 156480. 10.1016/j.phymed.2025.156480 39978273

[B93] Pan Y. Chen K. Hua W. Yu L. Bao W. Shi C. (2025b). Effectiveness and safety of suhuang zhike capsules in adults with asthma: a multicenter, randomized, double-blinded, placebo-controlled trial. Phytomedicine 141, 156478. 10.1016/j.phymed.2025.156478 40220421

[B94] Pandey D. Fulton D. J. (2011). Molecular regulation of NADPH oxidase 5 *via* the MAPK pathway. Am. J. Physiol. Heart Circ. Physiol. 300, H1336–H1344. 10.1152/ajpheart.01163.2010 21297032 PMC3075021

[B95] Pantano C. Ather J. L. Alcorn J. F. Poynter M. E. Brown A. L. Guala A. S. (2008). Nuclear factor-kappaB activation in airway epithelium induces inflammation and hyperresponsiveness. Am. J. Respir. Crit. Care Med. 177, 959–969. 10.1164/rccm.200707-1096OC 18263801 PMC2361423

[B96] Pelaia C. Vatrella A. Gallelli L. Lombardo N. Sciacqua A. Savino R. (2021). Role of p38 mitogen-activated protein kinase in asthma and COPD: pathogenic aspects and potential targeted therapies. Drug Des. Devel Ther. 15, 1275–1284. 10.2147/DDDT.S300988 33790539 PMC8001041

[B97] Peng S. Gao J. Liu W. Jiang C. Yang X. Sun Y. (2016). Andrographolide ameliorates OVA-Induced lung injury in mice by suppressing ROS-Mediated NF-κB signaling and NLRP3 inflammasome activation. Oncotarget 7, 80262–80274. 10.18632/oncotarget.12918 27793052 PMC5348318

[B98] Peng H. L. Huang W. C. Cheng S. C. Liou C. J. (2018). Fisetin inhibits the generation of inflammatory mediators in interleukin-1β-induced human lung epithelial cells by suppressing the NF-κB and ERK1/2 pathways. Int. Immunopharmacol. 60, 202–210. 10.1016/j.intimp.2018.05.004 29758489

[B158] Peng W. Xia Q. Zhang Y. Cao D. Zheng X. (2024). VEGF and EGFR signaling pathways are involved in the baicalein attenuation of OVA-induced airway inflammation and airway remodeling in mice. Respir. Res. 25 (1), 10. 10.1186/s12931-023-02637-6 38178132 PMC10765748

[B99] Plavsic A. Bonaci-Nikolic B. Milenkovic B. Miskovic R. Kusic N. Dimitrijevic M. (2024). Asthma inflammatory phenotypes: how can we distinguish them? J. Clin. Med. 13, 526. 10.3390/jcm13020526 38256660 PMC10816410

[B100] Pyo Y. Kwon K. H. Jung Y. J. (2024). Anticancer potential of flavonoids: their role in cancer prevention and health benefits. Foods 13, 2253. 10.3390/foods13142253 39063337 PMC11276387

[B101] Qin W. Tong X. Liang R. Tang K. Wu X. Jia Y. (2021). Preservation of mitochondrial homeostasis is responsible for the ameliorative effects of suhuang antitussive capsule on non-resolving inflammation *via* inhibition of NF-κB signaling and NLRP3 inflammasome activation. J. Ethnopharmacol. 271, 113827. 10.1016/j.jep.2021.113827 33460751

[B102] Qin Y. Yang J. Li H. Li J. (2024a). Recent advances in the therapeutic potential of nobiletin against respiratory diseases. Phytomedicine 128, 155506. 10.1016/j.phymed.2024.155506 38522319

[B103] Qin Z. Chen Y. Liu N. Wang Y. Su L. Liang B. (2024b). Mechanisms of bushenyiqi decoction in the treatment of asthma: an investigation based on network pharmacology with experimental validation. Front. Pharmacol. 15, 1361379. 10.3389/fphar.2024.1361379 38590639 PMC10999575

[B104] Rahman I. (2002). Oxidative stress and gene transcription in asthma and chronic obstructive pulmonary disease: antioxidant therapeutic targets. Curr. Drug Targets Inflamm. Allergy 1, 291–315. 10.2174/1568010023344607 14561194

[B105] Ray A. Kolls J. K. (2017). Neutrophilic inflammation in asthma and association with disease severity. Trends Immunol. 38, 942–954. 10.1016/j.it.2017.07.003 28784414 PMC5711587

[B106] Ray A. Das J. Wenzel S. E. (2022). Determining asthma endotypes and outcomes: complementing existing clinical practice with modern machine learning. Cell Rep. Med. 3, 100857. 10.1016/j.xcrm.2022.100857 36543110 PMC9798025

[B107] Ren N. Wang J. Gao J. N. Chen C. Z. Cai Y. L. Su L. M. (2024). Matrine attenuates mitochondrial fragmentation in ovalbumin-induced asthmatic mice by activating the AMPK/Nrf2 pathway. arXiv 38, 5329–5341.

[B108] Rizzi M. Barrella M. Kotzalidis G. D. Bevilacqua M. (2011). Periodic limbic movement disorder during sleep as diabetes-related syndrome? A polysomnographic study. ISRN Endocrinol. 2011, 246157. 10.5402/2011/246157 22363869 PMC3262626

[B109] Ruan G. Tao B. Wang D. Li Y. Wu J. Yin G. (2016). Chinese herbal medicine formula gu-ben-fang-xiao-tang attenuates airway inflammation by modulating Th17/Treg balance in an ovalbumin-induced murine asthma model. Exp. Ther. Med. 12, 1428–1434. 10.3892/etm.2016.3507 27588063 PMC4998120

[B110] Saha P. Durugkar S. Jain S. Shantanu P. A. Panda S. R. Jala A. (2022). Piperine attenuates cigarette smoke-induced oxidative stress, lung inflammation, and epithelial-mesenchymal transition by modulating the SIRT1/Nrf2 axis. Int. J. Mol. Sci. 23, 14722. 10.3390/ijms232314722 36499047 PMC9740588

[B111] Sahiner U. M. Birben E. Erzurum S. Sackesen C. Kalayci O. (2011). Oxidative stress in asthma. World Allergy Organ J. 4, 151–158. 10.1097/WOX.0b013e318232389e 23268432 PMC3488912

[B112] Sakkal M. Jarab A. S. Al Meslamani A. Z. (2026). Spatially resolved airway niches: mapping epithelial-immune microenvironments in asthma. Expert Rev. Respir. Med., 20 (3), 307–320. 10.1080/17476348.2025.2582244 41146609

[B113] Santibáñez M. Núñez-Robainas A. Barreiro E. Expósito A. Agüero J. García-Rivero J. L. (2025). Characterization of systemic oxidative stress in asthmatic adults compared to healthy controls and its association with the oxidative potential of particulate matter collected using personal samplers. Antioxidants (Basel) 14, 385. 10.3390/antiox14040385 40298638 PMC12024361

[B114] Saunders R. M. Biddle M. Amrani Y. Brightling C. E. (2022). Stressed out - the role of oxidative stress in airway smooth muscle dysfunction in asthma and COPD. Free Radic. Biol. Med. 185, 97–119. 10.1016/j.freeradbiomed.2022.04.011 35472411

[B115] Shan H. Li X. Ouyang C. Ke H. Yu X. Tan J. (2022). Salidroside prevents PM2.5-induced BEAS-2B cell apoptosis *via* SIRT1-dependent regulation of ROS and mitochondrial function. Ecotoxicol. Environ. Saf. 231, 113170. 10.1016/j.ecoenv.2022.113170 35026589

[B116] Shi P. Zhan Z. Ye X. Lu Y. Song K. Sheng F. (2022). The antioxidative effects of empagliflozin on high glucose-induced epithelial-mesenchymal transition in peritoneal mesothelial cells *via* the Nrf2/HO-1 signaling. Ren. Fail 44, 1528–1542. 10.1080/0886022X.2022.2118066 36098217 PMC9481091

[B117] Sies H. (2020). Oxidative stress: concept and some practical aspects. Antioxidants (Basel) 9 (9), 852. 10.3390/antiox9090852 32927924 PMC7555448

[B118] Soga M. Matsuzawa A. Ichijo H. (2012). Oxidative stress-induced diseases *via* the ASK1 signaling pathway. Int. J. Cell Biol. 2012, 439587. 10.1155/2012/439587 22654913 PMC3359665

[B119] Song B. Hu J. Chen S. Zhang Y. (2025). The mechanisms and therapeutic implications of PI3K signaling in airway inflammation and remodeling in asthma. Biologics 19, 73–86. 10.2147/BTT.S497622 40070559 PMC11895685

[B120] Stowe D. F. Camara A. K. (2009). Mitochondrial reactive oxygen species production in excitable cells: modulators of mitochondrial and cell function. Antioxid. Redox Signal 11, 1373–1414. 10.1089/ars.2008.2331 19187004 PMC2842133

[B121] Sun J. Li L. Wu J. Liu B. Gong W. Lv Y. (2013). Effects of baicalin on airway remodeling in asthmatic mice. Planta Med. 79, 199–206. 10.1055/s-0032-1328197 23378200

[B122] Sun Z. Ji N. Ma Q. Zhu R. Chen Z. Wang Z. (2020). Epithelial-mesenchymal transition in asthma airway remodeling is regulated by the IL-33/CD146 axis. Front. Immunol. 11, 1598. 10.3389/fimmu.2020.01598 32793232 PMC7387705

[B123] Sun Y. Han Y. Guo W. Xu X. Zhao L. Yang J. (2024). Multi-omics analysis of lung tissue metabolome and proteome reveals the therapeutic effect of shegan mahuang decoction against asthma in rats. J. Ethnopharmacol. 322, 117650. 10.1016/j.jep.2023.117650 38135230

[B124] Sussan T. E. Gajghate S. Chatterjee S. Mandke P. Mccormick S. Sudini K. (2015). Nrf2 reduces allergic asthma in mice through enhanced airway epithelial cytoprotective function. Am. J. Physiol. Lung Cell Mol. Physiol. 309, L27–L36. 10.1152/ajplung.00398.2014 25957295 PMC4491510

[B125] Suzuki T. Takahashi J. Yamamoto M. (2023). Molecular basis of the KEAP1-NRF2 signaling pathway. Mol. Cells 46, 133–141. 10.14348/molcells.2023.0028 36994473 PMC10070164

[B126] Teyani R. L. Moghaddam F. Moniri N. H. (2024). ROS-Mediated regulation of β2AR function: does oxidation play a meaningful role towards β2-agonist tachyphylaxis in airway obstructive diseases? Biochem. Pharmacol. 226, 116403. 10.1016/j.bcp.2024.116403 38945277 PMC11301793

[B127] Varricchi G. Brightling C. E. Grainge C. Lambrecht B. N. Chanez P. (2024). Airway remodelling in asthma and the epithelium: on the edge of a new era. Eur. Respir. J. 63, 2301619. 10.1183/13993003.01619-2023 38609094 PMC11024394

[B128] Wang J. Li F. Yang M. Wu J. Zhao J. Gong W. (2014). FIZZ1 promotes airway remodeling through the PI3K/Akt signaling pathway in asthma. Exp. Ther. Med. 7, 1265–1270. 10.3892/etm.2014.1580 24940423 PMC3991528

[B156] Wang M. H. Chen C. Yeh M. L. Lin J. G. (2019). Using traditional Chinese medicine to relieve asthma symptoms: a systematic review and meta-analysis. Am. J. Chin. Med. 47 (8), 1659–1674. 10.1142/S0192415X1950085X 31795745

[B129] Wang D. Chen J. Pu L. Yu L. Xiong F. Sun L. (2023a). Galangin: a food-derived flavonoid with therapeutic potential against a wide spectrum of diseases. Phytother. Res. 37, 5700–5723. 10.1002/ptr.8013 37748788

[B130] Wang D. Cui Y. Gao F. Zheng W. Li J. Xian Z. (2023b). Effects of imperatorin on airway remodeling in bronchial asthma through S1PR2/STAT3 signaling pathway. Cell Mol. Biol. (Noisy-le-grand) 69, 1–5. 10.14715/cmb/2023.69.15.1 38279507

[B131] Wang K. Wang L. Zhao G. Liu Y. Wang F. Song H. (2023c). Mechanistic study of salidroside on ovalbumin-induced asthmatic model mice based on untargeted metabolomics analysis. Food Funct. 14, 413–426. 10.1039/d2fo02225g 36515134

[B132] Wang X. Shi X. Xi Z. Zhang Z. Luo Z. Wang J. (2026). The scientific basis of synergy in traditional Chinese medicine: physicochemical, pharmacokinetic, and pharmacodynamic perspectives. Chin. Med. 21, 15. 10.1186/s13020-025-01291-y 41501914 PMC12781651

[B133] Wei Y. Luo Q. L. Sun J. Chen M. X. Liu F. Dong J. C. (2015). Bu-shen-yi-qi formulae suppress chronic airway inflammation and regulate Th17/Treg imbalance in the murine ovalbumin asthma model. J. Ethnopharmacol. 164, 368–377. 10.1016/j.jep.2015.01.016 25625352

[B134] Wei L. Hongping H. Chufang L. Cuomu M. Jintao L. Kaiyin C. (2023). Effects of shiwei longdanhua formula on LPS induced airway mucus hypersecretion, cough hypersensitivity, oxidative stress and pulmonary inflammation. Biomed. Pharmacother. 163, 114793. 10.1016/j.biopha.2023.114793 37121151

[B135] Winkler T. Frey U. (2021). Airway remodeling: shifting the trigger point for exacerbations in asthma. J. Allergy Clin. Immunol. 148, 710–712. 10.1016/j.jaci.2021.07.010 34310927

[B136] Xian Z. Choi Y. H. Zheng M. Jiang J. Zhao Y. Wang C. (2020). Imperatorin alleviates ROS-mediated airway remodeling by targeting the Nrf2/HO-1 signaling pathway. Biosci. Biotechnol. Biochem. 84, 898–910. 10.1080/09168451.2019.1710107 31900049

[B137] Xing Q. Q. Liu L. W. Zhao X. Lu Y. Dong Y. M. Liang Z. Q. (2019). Serum proteomics analysis based on label-free revealed the protective effect of Chinese herbal formula gu-ben-fang-xiao. Biomed. Pharmacother. 119, 109390. 10.1016/j.biopha.2019.109390 31520916

[B138] Xing Q. You Y. Zhao X. Ji J. Yan H. Dong Y. (2021). iTRAQ-Based proteomics reveals gu-ben-fang-xiao decoction alleviates airway remodeling *via* reducing extracellular matrix deposition in a murine model of chronic remission asthma. Front. Pharmacol. 12, 588588. 10.3389/fphar.2021.588588 34194321 PMC8237094

[B154] Xu M. Zhang D. Yan J. (2024). Targeting ferroptosis using Chinese herbal compounds to treat respiratory diseases. Phytomedicine 130, 155738. 10.1016/j.phymed.2024.155738 38824825

[B139] Xue-Jing Y. Yi-Qiu S. Su-Mei W. Yan-Ning L. I. Meng-Qing W. Xiao-Ran L. (2010). Clinical research of gubenfangxiao decoction combined with point application on chronic asthmatic children in 100 cases. China J. Traditional Chin. Med. Pharm. 2306–2309.

[B159] Ye P. Wu H. Jiang Y. Xiao X. Song D. Xu N. (2022). Old dog, new tricks: polydatin as a multitarget agent for current diseases. Phytother. Res. 36 (1), 214–230. 10.1002/ptr.7306 34936712

[B140] Yen M. H. Lee J. J. Yeh C. F. Wang K. C. Chiang Y. W. Chiang L. C. (2014). Yakammaoto inhibited human coxsackievirus B4 (CVB4)-induced airway and renal tubular injuries by preventing viral attachment, internalization, and replication. J. Ethnopharmacol. 151, 1056–1063. 10.1016/j.jep.2013.11.049 24361333

[B141] Yi L. Cui J. Wang W. Tang W. Teng F. Zhu X. (2020). Formononetin attenuates airway inflammation and oxidative stress in murine allergic asthma. Front. Pharmacol. 11, 533841. 10.3389/fphar.2020.533841 33013383 PMC7500463

[B142] You L. Yang C. Du Y. Wang W. Sun M. Liu J. (2020). A systematic review of the pharmacology, toxicology and pharmacokinetics of matrine. Front. Pharmacol. 11, 01067. 10.3389/fphar.2020.01067 33041782 PMC7526649

[B143] You Y. N. Xing Q. Q. Zhao X. Ji J. J. Yan H. Zhou T. (2021). Gu-ben-fang-xiao decoction modulates lipid metabolism by activating the AMPK pathway in asthma remission. Biomed. Pharmacother. 138, 111403. 10.1016/j.biopha.2021.111403 33714782

[B144] Yu Q. Shi Y. Shu C. Ding X. Zhu S. Shen Z. (2021). Andrographolide inhibition of Th17-Regulated cytokines and JAK1/STAT3 signaling in OVA-stimulated asthma in mice. Evid. Based Complement. Altern. Med. 2021, 6862073. 10.1155/2021/6862073 34194525 PMC8181172

[B145] Yuan K. Li X. Lu Q. Zhu Q. Jiang H. Wang T. (2019). Application and mechanisms of triptolide in the treatment of inflammatory Diseases-A review. Front. Pharmacol. 10, 1469. 10.3389/fphar.2019.01469 31866868 PMC6908995

[B146] Zeng H. Wang Y. Gu Y. Wang J. Zhang H. Gao H. (2019). Polydatin attenuates reactive oxygen species-induced airway remodeling by promoting Nrf2-mediated antioxidant signaling in asthma mouse model. Life Sci. 218, 25–30. 10.1016/j.lfs.2018.08.013 30092299

[B147] Zhai C. Wang D. (2022). Baicalin regulates the development of pediatric asthma *via* upregulating microRNA-103 and mediating the TLR4/NF-κB pathway. J. Recept Signal Transduct. Res. 42, 230–240. 10.1080/10799893.2021.1900865 33730981

[B148] Zhang K. Lu J. Mori T. Smith-Powell L. Synold T. W. Chen S. (2011). Baicalin increases VEGF expression and angiogenesis by activating the ERR{alpha}/PGC-1{alpha} pathway. Cardiovasc Res. 89, 426–435. 10.1093/cvr/cvq296 20851810 PMC3020130

[B149] Zhang C. Zhang L. H. Wu Y. F. Lai T. W. Wang H. S. Xiao H. (2016). Suhuang antitussive capsule at lower doses attenuates airway hyperresponsiveness, inflammation, and remodeling in a murine model of chronic asthma. Sci. Rep. 6, 21515. 10.1038/srep21515 26861679 PMC4748281

[B150] Zhang B. Feng X. Tian L. Xiao B. Hou L. Mo B. (2025). Epithelial-mesenchymal transition in asthma: its role and underlying regulatory mechanisms. Front. Immunol. 16, 1519998. 10.3389/fimmu.2025.1519998 39911398 PMC11794105

[B151] Zhou Y. Duan Q. Yang D. (2023a). *In vitro* human cell-based models to study airway remodeling in asthma. Biomed. Pharmacother. 159, 114218. 10.1016/j.biopha.2023.114218 36638596

[B152] Zhou Z. Qi J. Wu Y. Li C. Bao W. Lin X. (2023b). Nuciferine effectively protects mice against acetaminophen-induced liver injury. Antioxidants (Basel) 12, 949. 10.3390/antiox12040949 37107324 PMC10136285

[B153] Zhu X. Li Q. (2018). Epithelial mesenchymal transition in airway remodeling of asthma and its molecular regulation. Zhong Nan Da Xue Xue Bao Yi Xue Ban. 43, 566–570. 10.11817/j.issn.1672-7347.2018.05.016 29886474

